# Brain activity patterns underlying memory confidence

**DOI:** 10.1111/ejn.15649

**Published:** 2022-03-29

**Authors:** Syanah C. Wynn, Erika Nyhus

**Affiliations:** ^1^ Department of Psychology and Program in Neuroscience Bowdoin College Brunswick Maine USA

**Keywords:** decision making, episodic memory, medial temporal lobe, posterior parietal cortex, prefrontal cortex, recognition

## Abstract

The primary aim of this review is to examine the brain activity patterns that are related to subjectively perceived memory confidence. We focus on the main brain regions involved in episodic memory: the medial temporal lobe (MTL), prefrontal cortex (PFC), and posterior parietal cortex (PPC), and relate activity in their subregions to memory confidence. How this brain activity in both the encoding and retrieval phase is related to (subsequent) memory confidence ratings will be discussed. Specifically, encoding related activity in MTL regions and ventrolateral PFC mainly shows a positive linear increase with subsequent memory confidence, while dorsolateral and ventromedial PFC activity show mixed patterns. In addition, encoding‐related PPC activity seems to only have indirect effects on memory confidence ratings. Activity during retrieval in both the hippocampus and parahippocampal cortex increases with memory confidence, especially during high‐confident recognition. Retrieval‐related activity in the PFC and PPC show mixed relationships with memory confidence, likely related to post‐retrieval monitoring and attentional processes, respectively. In this review, these MTL, PFC, and PPC activity patterns are examined in detail and related to their functional roles in memory processes. This insight into brain activity that underlies memory confidence is important for our understanding of brain–behaviour relations and memory‐guided decision making.

## Graphical abstract

We have reviewed the neuroscientific literature to uncover the brain activity patterns associated with subjectively perceived memory confidence. Here, we show a summary of the activity patterns associated with the eight brain regions we investigated, separated by memory phase.

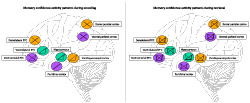

**Abbreviation List**
AtoM: attention to memory
BA: Brodmann area
BIC: binding of items in contexts
DLPFC: dorsolateral prefrontal cortex
DPC: dorsal parietal cortex
fMRI: functional magnetic resonance imaging
MTL: medial temporal lobe
NIBS: non‐invasive brain stimulation
PFC: prefrontal cortex
PPC: posterior parietal cortex
RK: remember/know
TMS: transcranial magnetic stimulation
tACS: transcranial alternating current stimulation
tDCS: transcranial direct current stimulation
VLPFC: ventrolateral prefrontal cortex
vmPFC: ventromedial prefrontal cortex
VPC: ventral parietal cortex

## 1 INTRODUCTION

The ability to retrieve previously experienced events from memory is supported by various brain regions. Specifically, regions in the medial temporal lobe (MTL), prefrontal cortex (PFC) and posterior parietal cortex (PPC) play a consistent role in memory‐related networks (Bastin et al., 2019; Benoit & Schacter, 2015; Nilakantan et al., 2017; Wang, Ranganath, & Yonelinas, 2014). Most of the literature on this type of memory, referred to as episodic memory, primarily gives insight on processes involved in ‘objective’ memory performance. In most memory studies, items are presented to the participants, which are to be intentionally or incidentally encoded. Subsequently, they are asked to retrieve these items from memory, usually by indicating whether they recognize the item as previously presented during the experiment (‘old’) or not (‘new’). In these cases, objective memory refers to the ability to distinguish between old and new items as a measure of item memory performance. Because it is known which items were presented during the encoding phase, the participants' ‘old/new’ response can be either true or false. In addition, participants are sometimes asked to recall certain details about the relationship between the encoded item and an additional experimentally manipulated aspect. In this way associative or source memory can be assessed. However, there is also a subjective aspect to memory, which often receives less attention. When you retrieve a memory, there is more to it than the dichotomy between true or false. False memories can feel very real, while true memories can feel doubtful. Understanding more about this subjective aspect is essential, given that this can have significant real‐life implications. For instance, witness confidence in the courtroom impacts their credibility and sentencing outcome (Brewer & Burke, 2002; Cramer et al., 2009). This subjective feeling about the trust we have in our memories can be measured with a subjective rating of memory confidence. It is important to note that there is no one‐to‐one mapping between objective memory performance and memory confidence. There can be a positive, negative, or no correlation between both, indicating two different processes (Chua et al., 2012; DeSoto & Roediger, 2014; Loftus & Pickrell, 1995; Muller et al., 2021; Pena et al., 2017).

It is important to gain more knowledge about self‐reported memory confidence because this adds another dimension to old/new judgements, making it possible to investigate more subtle brain‐behaviour relations. It has been shown that reductions in subjective memory can be an early sign of brain disorders, like dementia, which will not easily be detected with standard tests used in clinical practice (Metzler‐Baddeley et al., 2012; Mitchell et al., 2014; Oijen et al., 2007). This underlines the relevance of more knowledge on the brain processes involved in subjectively perceived memory confidence. Memory confidence is closely related to two recognition memory processes: recollection and familiarity. Recollection reflects the retrieval of qualitative information about an event and is frequently accompanied by a relatively high memory confidence. On the other hand, familiarity is hypothesized to reflect levels of linear memory strength and often is accompanied by lower memory confidence (Yonelinas, 2002). Memory confidence is the output of decision making processes occurring during memory retrieval. During these processes, there is a criterion that determines whether a person calls an item ‘old’ or ‘new’, controlling objective memory performance. In addition, the retrieved information is being validated and compared with the current goals, leading to a graded scale of memory confidence. This is based on the amount of information retrieved from memory, and the quality and relevance of this information. Even when an item is recognized as old, because it passed the ‘old criterion’, it could still be that the person is not completely sure about their memory. This memory confidence will influence decision making in real life, where we utilize our memory to guide our day to day behaviour.

**FIGURE 1:**
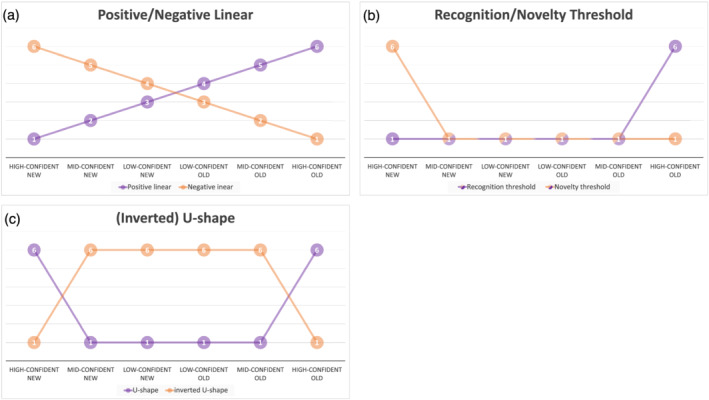
Confidence patterns. (a) Linear relationships with memory confidence. Either a linear increase in activity with memory confidence (positive linear) or a linear decrease in activity with memory confidence (negative linear). (b) Threshold‐based relationships with memory confidence. Either a sharp increase in activity for high‐confident old (recognition threshold) or high‐confident new (novelty threshold) items. (c) U‐shaped relationships with memory confidence. Either a peak in activation during high‐confident responses (U‐shape) or a peak in activation during low‐confident responses (inverted U‐shape)

Here, we will review the neuroscience literature on subjectively perceived memory confidence. Specifically, we will focus on activity patterns in brain regions known to be involved in episodic memory processing (MTL, PFC, and PPC) and uncover their involvement in memory confidence. We will start our discussion introducing encoding‐related brain activity related to subsequent memory confidence. Whether we can confidently remember having encountered an item, partly depends on the encoding of that item. When information is encoded successfully, the chances of a confident recognition greatly increase. Therefore, specific encoding‐related brain activity might be able to predict later memory confidence. Thereafter, we will shift our attention to retrieval‐related activity related to memory confidence. Because memory confidence is a continuous construct that is often measured by more than two levels, we not only focus on regions that show a sensitivity to memory confidence, but also describe specific activity patterns. When only one or two levels of confidence are reported (e.g., high and low confidence), we describe this, but these studies were not assigned a specific activity pattern. Because the confidence ratings in the studies we discuss often go from high‐confident new to high‐confident old, the brain can show a linear increase or decrease in activity when memory confidence increases (positive linear or negative linear; see Figure 1a), a sharp increase specifically to high‐confident old or high‐confident new responses (recognition threshold or novelty threshold; see Figure 1b), or a selective response to high‐ or low‐confident responses, irrespective of memory status (U‐shape or inverted U‐shape; see Figure 1c). We will use these encoding‐ and retrieval‐related activity patterns to describe the specific role of MTL, PFC, and PPC regions in memory confidence. Figure 1 serves as an illustration of these patterns with six levels. Studies discussed here vary in number of levels and labels used to define those levels. We have grouped each result from the available literature in one of the six predefined activity patterns (see Tables 1 and 2). See Tables 3 and 4 for more details on the memory tasks, confidence ratings, and activation patterns for the studies reviewed below. In this way, we hope to get a more dynamic picture of the brain regions involved in subjectively perceived memory confidence.

**TABLE 1:** Overview of the number of encoding findings per brain region and activity pattern

	Hippocampus	Parahippocampal cortex	Perirhinal cortex	Dorsolateral PFC	Ventrolateral PFC	Ventromedial PFC	Ventral parietal cortex	Dorsal parietal cortex
Positive linear	3	1	4	3	2	1	3	1
Negative linear	0	0	0	1	0	2	3	2
Recognition threshold	1	1	0	0	2	1	0	0

**TABLE 2:** Overview of the number of retrieval findings per brain region and activity pattern

	Hippocampus	Parahippocampal cortex	Perirhinal cortex	Dorsolateral PFC	Ventrolateral PFC	Ventromedial PFC	Ventral parietal cortex	Dorsal parietal cortex
Positive linear	3	3	2	6	5	4	6	8
Negative linear	1	1	2	2	3	3	1	2
Recognition threshold	11	2	0	1	0	3	2	1
Novelty threshold	0	0	0	0	0	0	0	0
U‐shape	4	1	2	1	2	4	4	0
Inverted U‐shape	0	0	2	2	3	1	1	2

**TABLE 3:** Summary of encoding‐related confidence patterns reported in the literature

Article	Memory task	Analysed confidence levels	Hippocampus	Parahippocampal cortex	Perirhinal cortex	Dorsolateral PFC	Ventrolateral PFC	Ventromedial PFC	Ventral parietal cortex	Dorsal parietal cortex
Kirwan et al. (2008)	Word (item) animate/shoebox judgement (source) memory task	(1) Sure new, (2) probably new, (3) guess new, (4) guess old, (5) probably old, (6) sure old	Positive linear		Positive linear	Positive linear			Negative linear	
Qin et al. (2011)	Description of a scene (item) memory task	VAS scale & (1) unsure/somewhat sure/sure, (2) very sure					Positive linear Recognition threshold		Positive linear	
Shrager et al. (2008)	Word (item) memory task	(1) Definitely/probably new, (2) maybe new, (3) maybe old, (4) probably old, (5) definitely old	Positive linear		Positive linear	Negative linear		Negative linear	Negative linear	Negative linear
Sommer et al. (2005)	Picture (item) spatial (source) memory task	(1) Forgotten location, (2) four locations, including correct one, (3) three locations, including correct one, (4) two locations, including correct one, (5) one correct location		Positive linear		Positive linear	Positive linear	Positive linear	Positive linear	Positive linear
Song, Jeneson, and Squire (2011)	Word (item) scene (source) memory task	(1) Definitely/probably new, (2) guess new, (3) guess old, (4) probably old, (5) definitely old & (1) low‐strength source memories, (2) medium‐strength source memories, (3) high‐strength source memories	Positive linear		Positive linear	Positive linear		Negative linear	Positive linear Negative linear	Negative linear
Song, Wixted, et al. (2011)	Word (item) memory task	(1) Misses, (2) low confident hits, (3) medium confident hits, (4) high confident hits	Recognition threshold	Recognition threshold	Positive linear		Recognition threshold	Recognition threshold		

**TABLE 4:** Summary of retrieval‐related confidence patterns reported in the literature

Article	Memory task	Analysed confidence levels	Hippocampus	Parahippocampal cortex	Perirhinal cortex	Dorsolateral PFC	Ventrolateral PFC	Ventromedial PFC	Ventral parietal cortex	Dorsal parietal cortex
Cohn et al. (2009)	Word‐pair (item) memory task	(1) Sure new, (2) unsure new, (3) unsure familiar, (4) sure familiar, (5) recollect	Recognition threshold			Positive linear	Positive linear		Positive linear	Recognition threshold
Daselaar, Fleck, Dobbins, et al. (2006)	Word (item) memory task	(1) New, (2) probably old, (3) definitely old	Recognition threshold				Negative linear	Negative linear Recognition threshold	U‐shape	Negative linear
Daselaar, Fleck, and Cabeza (2006)	Word (item) memory task	(1) Definitely new, (2) probably new, (3) maybe new, (4) maybe old, (5) probably old, (6) definitely old	Recognition threshold Negative linear	Positive linear		Negative linear	Negative linear U‐shape	Positive linear Negative linear Recognition threshold	U‐shape	Positive linear
Hou et al. (2021)	Word (item) memory task	(1) Confident new, (2) unconfident new, (3) unconfident old, (4) confident old, (5) recollect	U‐shape	U‐shape	U‐shape	Positive linear		U‐shape	U‐shape Recognition thresholds	Positive linear
Hutchinson et al. (2015)	Word (item) memory task	(1) High confident new, (2) low confident new, (3) unconfident, (4) low confident old (5) high confident old				Positive linear U‐shape Inverted U‐shape	Positive linear U‐shape Inverted U‐shape	U‐shape	Positive linear Inverted U‐shape U‐shape	Positive linear Inverted U‐shape
Johnson et al. (2013)	Word (item) scene/sentence (source) memory task	(1) Confident new, (2) unconfident new, (3) unconfident old, (4) confident old, (5) remembered	Recognition threshold U‐shape	Recognition threshold		Positive linear Inverted U‐shape	Inverted U‐shape	Positive linear Recognition threshold Inverted U‐shape	Recognition threshold	Positive linear
Kafkas et al. (2017)	Scene/object/face (item) memory task	(1) Miss, (2) weak familiarity, (3) moderate familiarity, (4) strong familiarity (5) recollection	Recognition threshold	Positive linear Negative linear	Positive linear Negative linear					
Kafkas and Montaldi (2012)	Picture (item) memory task	(1) Correct rejection, (2) miss, (3) weakly familiar, (4) moderately familiar, (5) strongly familiar, (6) recollect	Recognition threshold U‐shape		Inverted U‐shape	Positive linear Recognition threshold	Inverted U‐shape		Positive linear	
Kirwan et al. (2009)	Word (item) memory task	(1) Sure new, (2) probably new, (3) guess new, (4) guess old, (5) probably old, (6) probably new	Positive linear Recognition threshold							
Mayes et al. (2019)	Word (item) memory task & word cued recall	(1) Miss, (2) weak familiarity, (3) moderate familiarity, (4) strong familiarity & (1) miss, (2) weak recollection, (3) moderate recollection, (4) strong recollection	Positive linear			Positive linear	Positive linear	Positive linear	Positive linear	Positive linear
Montaldi et al. (2006)	Scene (item) memory task	(1) Correct rejection, (2) miss, (3) very weakly familiar, (4) moderately familiar, (5) strongly familiar, (6) recollect	Recognition threshold		Negative linear	Negative linear	Positive linear Negative linear		Positive linear	Positive linear
Moritz et al. (2006)	Word (item) memory task	(1) 100% confident correct rejection, (2), rather confident correct rejection, (3) guess correct rejection, (4) guess hit, (5) rather confident hit, (6) 100% confident hit						U‐shape		Inverted U‐shape
Mugikura et al. (2010)	Picture (item) and male/female (source) memory task	(1) Correct rejection, (2) miss, (3) low confident source, item‐only hit, (4) low confident source, source and item hit, (5) high confident source, source and item hit	U‐shape		Inverted U‐shape					
Slotnick and Thakral (2013)	Shape (item) and left/right (source) memory task	(1) Sure source miss, (2) unsure source miss, (3) unsure source hit, (4) sure source hit	Recognition threshold							
Yonelinas et al. (2005)	Word (item) memory task	(1) Entirely sure new, (2) not entirely sure new, (3) not entirely sure old, (4) entirely sure old, (5) remember	Recognition threshold	Recognition threshold			Positive linear	Positive linear Negative linear U‐shape	Positive linear	Positive linear
Wang, Ranganath, and Yonelinas (2014)	Word (item) memory task	(1) Sure new, (2) bit sure new, (3) unsure new, (4) unsure old, (5) bit sure old, (6) very sure old	Recognition threshold		U‐shape					
Woroch et al. (2019)	Word (item) and face/scene (source) memory task	(1) Guess source, (2) think source, (3) sure source	Positive linear	Positive linear	Positive linear				Negative linear	Positive linear Negative linear

As can be seen in Tables 3 and 4, there is variability in the confidence ratings used across studies, making it difficult to directly compare activity patterns between studies. Especially because participants are making a subjective response, their responses can be influenced by the labels and number of categories used by the researchers. We anticipate that this will have a small effect on the high‐confidence responses, because those are on the extremes of the scale, regardless of labels or number of response options. However, it may have a bigger effect on the lower‐confidence responses, because the internal threshold between, for example, ‘not sure’, ‘a bit sure’, ‘maybe’, and ‘probably’ may be less clear and thus leads to more variability. To standardize this, we opted to categorize findings from the discussed literature into six categories based on activity patterns found. Not all ratings allowed us to make a clear categorization in our six activity pattern options. In those cases, we chose the one that seemed most plausible. Even though there is a subjective aspect to the classifications made, we believe that this approach enabled us to look at the bigger picture. Assessing the specific relationship between brain activity in memory‐related areas and confidence ratings. It is beyond the scope of this review to give a comprehensive overview of the literature on ‘Remember/Know’ (RK) paradigms, source judgements, receiver operator characteristic (ROC) curves, and the relations between those. We refer interested readers to the following literature: Aggleton and Brown (2006); Skinner and Fernandes (2007); Squire et al. (2007); Yonelinas and Parks (2007); Mitchell and Johnson (2009); Spaniol et al. (2009); Rugg and Vilberg (2013).

The review will focus mainly on studies with healthy adult participants using functional magnetic resonance imaging (fMRI) to map out brain activity in specified predefined brain areas. Moreover, given the recent increase in papers using non‐invasive brain stimulation (NIBS) techniques, we are also able to move from correlational to causal conclusions about the involvement of brain regions. These NIBS techniques, including transcranial magnetic stimulation (TMS), transcranial direct current stimulation (tDCS), and transcranial alternating current stimulation (tACS), can be used to enhance or reduce neuronal excitability, or to entrain brain activity to a specific frequency. Because of the focus on brain regions involved in subjective memory in healthy adults, this review will not directly discuss literature on patient, electrophysiological, or behavioural studies, although references to studies utilizing these methods are made where relevant. We refer interested readers to other sources for more extensive coverage of patient (Hoven et al., 2019; Muller & Roberts, 2005; Schnyer et al., 2004; Simons et al., 2010), electrophysiological (Addante et al., 2012; Muller et al., 2021; Reed et al., 2020; Wynn et al., 2019; Wynn et al., 2020), and behavioural (Shing et al., 2009; Yonelinas & Parks, 2007) studies.

## 2 ENCODING‐RELATED BRAIN ACTIVITY

### 2.1 Medial temporal lobe

The MTL, including the hippocampus, parahippocampal cortex, and entorhinal cortex, is essential for episodic memory (Bastin et al., 2019; Eichenbaum et al., 2007; Preston & Eichenbaum, 2013; Rey et al., 2018; Schacter & Wagner, 1999; Solomon et al., 2019). The MTL is involved in both memory encoding, the transfer of information into a memory trace, and retrieval, reactivation of memory traces to access previously encoded information. Of all MTL regions, the hippocampus is the most studied and most consistently associated with memory functions. Specifically, neuropsychological lesion studies have shown the importance of the hippocampus by reporting that hippocampal damage leads to deficits in acquiring novel information (Hopkins et al., 1995; Milner, 1972; Scoville & Milner, 1957). However, it is difficult to dissociate between encoding‐ and retrieval‐related processing in lesion studies. Functional neuroimaging studies have shown that MTL activity during encoding appears to be related to spatial and temporal binding of events, which is especially important for associative memory (Eichenbaum et al., 2012; Jackson & Schacter, 2004; Rugg et al., 2012). For instance, encoding‐related activity in both the hippocampus and parahippocampal cortex is important to establish connections between memory elements, supporting associative memory (Henke et al., 1997; Jackson & Schacter, 2004; Jenkins & Ranganath, 2010; Kirwan et al., 2008; Kirwan & Stark, 2004; Tubridy & Davachi, 2011). On the other hand, it has been suggested that the perirhinal cortex is mainly involved in item memory (Kirwan & Stark, 2004; Rugg et al., 2012), although there have been reports of a relationship with subsequent successful associative memory as well (Awipi & Davachi, 2008).

Given the role of the MTL, especially the hippocampus, in forming associations between memory elements, it is expected that this brain area also plays a role in reported memory confidence. It is conceivable that more elaborate encoding would lead to higher subjectively perceived memory confidence at retrieval, because more information can be retrieved. Indeed, encoding‐related activity in the hippocampus seems to be the highest for subsequent high‐confident hits (Kirwan et al., 2008; Otten et al., 2001; Shrager et al., 2008; Song, Jeneson, & Squire, 2011; Song, Wixted, et al., 2011). This relationship between hippocampal activity and subsequent memory confidence mainly shows a positively linear pattern (see Figure 1a) (Kirwan et al., 2008; Shrager et al., 2008; Song, Jeneson, & Squire, 2011), but can also be a recognition threshold pattern, with the highest activity for high‐confident old responses and equivalent moderate activity for lower levels of confidence (see Figure 1b) (Song, Wixted, et al., 2011). Hippocampal activity during encoding thus appears to be beneficial for the ability to recognize items later with high confidence.

Likewise, encoding‐related activity in the parahippocampal cortex also increases with subsequent memory confidence, showing either a positive linear or a recognition threshold pattern (Sommer et al., 2005; Song, Wixted, et al., 2011). In a study where associative memory confidence was assessed parametrically, parahippocampal cortex activity showed a positive linear relationship with subsequent memory confidence (Sommer et al., 2005). On the other hand, the parahippocampal cortex showed a recognition threshold‐based activity pattern identical to the hippocampus when item memory confidence was measured (Song, Wixted, et al., 2011). The tasks used in these studies varied considerably, so the specific response pattern of the parahippocampal cortex could be task dependent. Given the limited number of studies reporting parahippocampal confidence response patterns, additional research is needed to specify the relationship between activity in parahippocampal cortex and memory confidence.

Finally, activity in the perirhinal cortex shows a consistent positive linear relationship with memory confidence (Kirwan et al., 2008; Shrager et al., 2008; Song, Jeneson, & Squire, 2011; Song, Wixted, et al., 2011), which can flatten at higher levels of memory confidence (Song, Wixted, et al., 2011). Like the hippocampus, encoding‐related activity in the perirhinal cortex thus seem to lead to higher subsequent memory confidence. The hippocampus and the perirhinal cortex could be working in tandem to optimize encoding (Brown & Aggleton, 2001). The perirhinal cortex and specifically the anterior hippocampus are functionally connected (Libby et al., 2012; Maass et al., 2015; Ritchey et al., 2015), possibly to enable processing and storing of information of memory items (Ritchey et al., 2015). For instance, both the hippocampus and perirhinal cortex show enhanced encoding activity for items that were consistently remembered over a period of time (Carr et al., 2010). When encoding is successful, there will be more evidence available for retrieval during the later memory recognition decision making process. This in turn will increase subjectively perceived confidence in the recognition.

It is worth noting that two studies that investigated associative memory confidence while keeping item memory confidence constant showed that none of the MTL regions showed a positive relationship with associative memory confidence (Kirwan et al., 2008; Song, Jeneson, & Squire, 2011). So, it could be that MTL related activity found for associative memory confidence is often confounded by its relation to item memory confidence. Nevertheless, it seems that all the MTL regions are positively related to memory confidence, where the hippocampus, and possibly the parahippocampal cortex, shows a specific sensitivity to high‐confident recognition.

### 2.2 Prefrontal cortex

The PFC is a well‐known node for cognitive control functions, which are required for episodic memory and might be especially important for generating memory confidence (Blumenfeld & Ranganath, 2007; Preston & Eichenbaum, 2013). During memory formation, cognitive control is needed for top‐down attention guiding the encoding of relevant information. Specifically, the ventrolateral PFC (VLPFC; BA44/45/47) is involved in the controlled selection of task‐relevant information during memory encoding, which supports subsequent memory for both item and associative memory (Blumenfeld et al., 2011; Blumenfeld & Ranganath, 2007). The role of the dorsolateral PFC (DLPFC; BA9/46) is less clear, with some studies showing a positive relationship between encoding‐related DLPFC activity and subsequent memory performance (Murray & Ranganath, 2007; Qin et al., 2007), whereas others do not (Daselaar et al., 2004; Otten & Rugg, 2001). It has been proposed that this discrepancy might be caused by a specific role for the DLPFC in the processing of distinct relationships during memory formation, which strengthens the associative information in long‐term memory (Blumenfeld et al., 2011; Blumenfeld & Ranganath, 2007). The contribution of the ventromedial PFC (vmPFC; BA10/11/12/24/25/32) to memory encoding is thought to be specific to instances when there is a match between the novel information and prior knowledge (Brod & Shing, 2018). The vmPFC seems to be critical to the application of prior knowledge to novel events, supporting associative memory (Spalding et al., 2018).

Based on the roles of these PFC regions in controlled and associative encoding, a positive relationship with subjective memory confidence is expected. Supporting this, greater VLPFC activity is related to high‐confident memory (Floel et al., 2004; Hales & Brewer, 2011; Kohler et al., 2004; Otten et al., 2001; Qin et al., 2011; Sommer et al., 2005). Functional imaging studies have mainly found effects in the left VLPFC, where activity shows a positively linear (Qin et al., 2011; Sommer et al., 2005) or recognition threshold (Qin et al., 2011; Song, Wixted, et al., 2011) relationship with subsequent memory confidence, which is stronger for the encoding of associative information (Hales & Brewer, 2011). Furthermore, TMS studies suggest a lateralisation in the roles the left and right VLPFC play in memory encoding. Subsequent confidence in verbal memories is enhanced when left VLPFC excitability is increased (Kohler et al., 2004) and decreasing this excitability reduces subsequent confidence in verbal memories (Floel et al., 2004; Kahn et al., 2005). This is evidence for an important role for the left VLPFC in the encoding of verbal information that can later be remembered with high confidence. In addition, subsequent memory confidence in abstract visual memories is reduced after the right VLPFC is inhibited (Floel et al., 2004). This suggests that encoding‐related activity in the right VLPFC is beneficial for memory confidence for visual information. Interestingly, inhibitory TMS over the right VLPFC also increased the subsequent accuracy of low‐confident verbal memories (Kahn et al., 2005). Thus, inhibiting this right‐lateralised area supports the encoding of weaker verbal memories, possibly by inhibiting task‐irrelevant visual processes.

Similar to VLPFC activity, both TMS (Demeter et al., 2016) and fMRI (Hales & Brewer, 2011; Kirwan et al., 2008; Sommer et al., 2005; Song, Jeneson, & Squire, 2011) studies show that DLPFC activity can be beneficial for subsequent memory confidence. Specifically, this relationship appears to be mainly positive linear (Kirwan et al., 2008; Sommer et al., 2005; Song, Jeneson, & Squire, 2011) and stronger for high‐confident associative than item hits (Hales & Brewer, 2011). Moreover, Demeter et al. (2016) showed in two experiments that TMS to facilitate encoding‐related left DLFPC activity led to a higher proportion of high‐confident hits. However, a negative linear relationship has been reported between right DLPFC activity and memory confidence (Shrager et al., 2008). This is in concordance with facilitatory TMS to the right DLPFC leading to reduced high‐confident word recognition (Kohler et al., 2004). This indicates a possible lateralisation in the role of the DLPFC in subsequent memory confidence, with left lateralised activity increasing (Demeter et al., 2016; Kirwan et al., 2008; Song, Jeneson, & Squire, 2011) and right lateralized activity decreasing subsequent memory confidence (Kohler et al., 2004; Shrager et al., 2008).

Fewer studies have reported a link between encoding‐related vmPFC activity and subsequent memory confidence. In some of these studies, there is a positive association between left vmPFC activity and memory confidence, which is either positively linear (Sommer et al., 2005) or shows a recognition threshold‐based pattern (Song, Wixted, et al., 2011). In others, a negative linear relationship between the vmPFC and memory confidence was found (Shrager et al., 2008; Song, Jeneson, & Squire, 2011). Based on these studies, the relationship between encoding‐related vmPFC activity and subsequent memory confidence appears to be mixed.

To summarize the above findings, there appears to be a lateralization within the PFC regarding the effect that encoding‐related activity has on subsequent memory confidence. This lateralization in the VLPFC is mainly influenced by stimulus material, with the left hemisphere involved in verbal encoding and the right one in non‐verbal encoding. On the other hand, lateralization in the DLPFC is reflected by a positive relationship in the left hemisphere and a negative one in the right hemisphere. The vmPFC activity does not appear to show a clear lateralisation, but additional research is needed to clarify the role of vmPFC in memory confidence.

### 2.3 Posterior parietal cortex

Although there is ample evidence linking PPC activity to episodic memory (Bjekic et al., 2019; Rubinstein et al., 2021; Sestieri et al., 2017; Spaniol et al., 2009; Wagner et al., 2005), its functional role remains elusive. First, the PPC has been implicated in the binding of elements into one episodic event. For instance, successful associative memory encoding is related to PPC activity (Hales & Brewer, 2013; Tibon et al., 2019; Uncapher et al., 2006) and NIBS techniques, like tACS and tDCS, targeting the PPC can alter subsequent associative memory performance (Meng et al., 2021; Vulic et al., 2021). Second, because the PPC has often been associated with the neurobiological underpinnings of attention, it has also been proposed that memory related PPC activity is modulated by attentional processes. Top‐down attention during encoding, mediated by the dorsal PPC, increases the probability of subsequent successful memory. The opposite holds for bottom‐up attention during encoding, mediated by the ventral PPC, which increases the likelihood that the item will be forgotten (Daselaar et al., 2009; Uncapher et al., 2006, 2011; Uncapher & Wagner, 2009), with the ventral parietal cortex (VPC) consisting of the angular gyrus (BA39), supramarginal gyrus (BA40), and the temporoparietal junction (BA39/40), and the dorsal parietal cortex (DPC) consisting of the superior parietal lobe (BA7), precuneus (BA7), and the intraparietal sulcus (BA7/39/40). Third, it is unclear whether the PPC has a direct or causal influence on memory encoding, because there is evidence that interfering with PPC activity does not necessarily alter subsequent memory performance (Dubravac & Meier, 2021; Meng et al., 2021; Rossi et al., 2006). In these studies, various NIBS techniques were applied over PPC regions while participants were encoding information, and results showed that this had no effect on subsequent memory performance. This could indicate that encoding‐related PPC effects found in the neuroimaging literature are only correlational in nature and might reflect indirect processes.

Functional neuroimaging literature on VPC and DPC activity, and subsequent memory confidence is sparse, but there are some indications regarding their involvement. A negative linear relationship has been reported between both VPC (Kirwan et al., 2008; Shrager et al., 2008; Song, Jeneson, & Squire, 2011) and DPC (Shrager et al., 2008; Song, Jeneson, & Squire, 2011) activity, and subsequent memory confidence. It has been suggested that this activity might represent task‐irrelevant mind wondering which is harmful to successful encoding (Shrager et al., 2008). On the other hand, Sommer et al. (2005) presented pictures to one of 16 locations on the screen during encoding and subsequently asked participants to select the remembered location of the picture in the encoding phase. Interestingly, participants were instructed to select as many locations as needed when they were unsure about the exact encoding‐location of the item. The number of selected locations was used as a measure of associative memory confidence. Activity in regions of both VPC and DPC showed a positive correlation with associative memory confidence. Later studies replicated this, by showing that left VPC activity showed a positive linear relation to subjectively perceived associative memory strength (Qin et al., 2011; Song, Jeneson, & Squire, 2011). Therefore, it appears that the involvement of PPC regions can be beneficial or harmful during encoding, possibly dependent on encoding conditions or task instructions.

Additionally, in the NIBS literature there appears to be some evidence that modulating VPC activity alters subsequent memory confidence (Koen et al., 2018; Tambini et al., 2018). For instance, Tambini et al. (2018) showed that inhibitory TMS over the right angular gyrus, enhanced associative memory confidence. However, the authors argue that this behavioural effect is not due to processes directly influenced by the angular gyrus, but through functional connections with the hippocampus. Another TMS study showed that when the left angular gyrus is inhibited, the reported confidence in erroneous associative memory is altered (Koen et al., 2018). Specifically, the confidence in associative misses is reduced, while confidence in associative false alarms is increased. Nevertheless, this study did not show an effect of angular gyrus TMS on confidence ratings regarding correct associative memories. Next to these mixed effects, there have been multiple NIBS studies that failed to find evidence for an important role for the PPC in subsequent memory confidence (Alekseichuk et al., 2020; Jacobson et al., 2012; Kohler et al., 2004). For instance, Alekseichuk et al. (2020) used EEG and fMRI to determine the optimal placement (parietal) and frequency (theta) for tACS stimulation. Their results showed that the left parietal cortex is involved in memory encoding, but has no effect on subsequent memory confidence (Alekseichuk et al., 2020).

It thus seems, based on these studies, that there is little evidence supporting the notion that encoding‐related PPC activity has a significant influence on subsequent memory confidence. Nevertheless, it appears that the effects might be stronger on associative memory as compared with item memory. As discussed before, the effects we see from the PPC might be due to connectivity with other regions, like the hippocampus, that are involved in associative encoding. Perhaps the PPC merely plays a supportive role in these networks, leading to inconsistent results.

### 2.4 Overall effects on memory confidence

**FIGURE 2:**
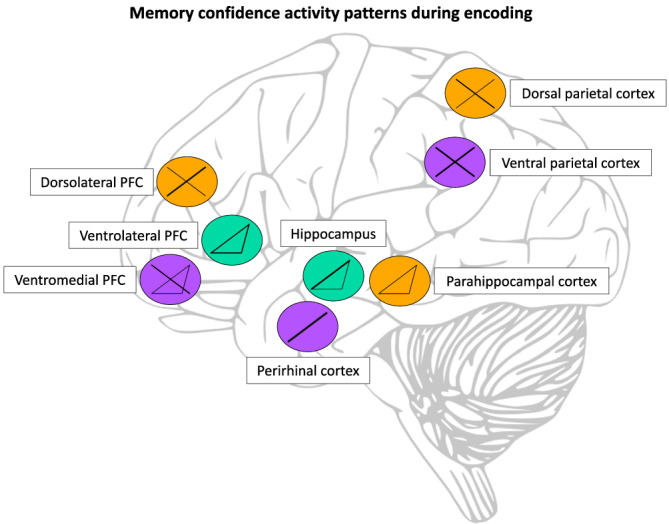
Summary of the encoding‐related activity patterns in relation to confidence levels in the relevant brain regions. Line thickness is scaled according to the number of studies supporting the pattern

The encoding‐related confidence activity patterns in the MTL, PFC, and PPC shed light upon their possible role in memory confidence during subsequent retrieval (see Figure 2 and Table 1). In MTL regions, we see that there is mainly a positive linear relationship between activity and subsequent memory confidence. In addition, the hippocampus and parahippocampal cortex can show a selective response for subsequent high‐confident memories. These patterns might result from their roles in the binding of separate memory features into one event (Eichenbaum et al., 2012; Jackson & Schacter, 2004; Rugg et al., 2012). The more details that are initially bound into a separate event, the more details are later available to signal higher confidence. Interestingly, because the same patterns are found in item and source tasks, the introduction of an explicit encoding source seems to have no influence on the patterns. Nevertheless, the encoding tasks the participants were performing would generate several memory features to be combined in the encoding event. Given its proposed role in item memory (Kirwan & Stark, 2004; Rugg et al., 2012), it is of interest that the perirhinal cortex does not show the threshold‐like increase in activity for high‐confident memories, like the other MTL regions. Perhaps lower levels of confidence can be attained without elaborate binding during encoding, while this binding is especially important to the highest level of confidence.

When we shift our focus to the PFC, we see that only the VLPFC shows a consistent positive relationship with later memory confidence. This indicates that, as predicted, VLPFC‐mediated top‐down control during memory encoding is beneficial for memory confidence (Blumenfeld et al., 2011; Blumenfeld & Ranganath, 2007). Focusing attention on relevant information supports the MTL regions with encoding of the event, contributing to later memory confidence. On the other hand, the DLPFC and vmPFC show inconsistent and even contrasting confidence related brain activity patterns across studies. This suggests that activity in these brain regions does not play a direct role in subsequent memory confidence, or that their specific role is lateralized or dependent on task qualities. For instance, both the DLPFC and vmPFC have been linked to the binding of elements, but only in specific conditions (Blumenfeld et al., 2011; Blumenfeld & Ranganath, 2007; Brod & Shing, 2018). Specifically, the DLPFC seems to be of particular importance when explicit relationships between encoding‐elements are formed (Blumenfeld et al., 2011), and there seems to be a lateralisation in the direction of the reported effects. Both negative findings concerning the right DLPFC were reported in studies using item memory tasks, and positive findings in the left DLPFC were mainly found in source memory tasks. Therefore, the left DLPFC seems to be important for the formation of associations between encoded elements, which may not significantly affect subsequent memory confidence in item memory tasks. On the other hand, the right DLPFC seems to be involved in processes that are harmful to later item memory confidence. What the specific nature of these processes is difficult to pinpoint given the limited number of studies reporting these effects. Encoding‐related vmPFC activity seems to be needed when novel to‐be‐encoded information matches prior knowledge (Brod & Shing, 2018). The variability in confidence‐related vmPFC patterns may therefore be explained by differences in tasks used across studies. Nevertheless, this indicates that the right DLPFC and vmPFC only play an indirect role in memory confidence, through specific processes that are not always needed and can even be harmful.

The inconsistent patterns in the PPC might be explained by its proposed indirect role through binding and attentional processes. Specifically, DPC‐mediated top‐down attention is beneficial for successful encoding, while VPC‐mediated bottom‐up attention decreases the chances for successful encoding (Daselaar et al., 2009; Uncapher et al., 2006, 2011; Uncapher & Wagner, 2009). This would predict that the DPC would show a positive pattern, while the VPC shows a negative pattern. However, both patterns were found in both regions. Perhaps attentional demands have an influence on the relationship with subsequent memory confidence. For instance, bottom‐up attention might be beneficial for the encoding of salient stimuli that may be easily linked to a participant's previous experiences, while it is harmful when it impairs the encoding of information that requires more top‐down control. Nonetheless, the null findings in NIBS studies indicate that there is no support for the notion that the PPC activity during encoding has a causal or direct relationship with subsequent memory confidence.

## 3 RETRIEVAL‐RELATED BRAIN ACTIVITY

### 3.1 Medial temporal lobe

It is proposed that after initial item binding during encoding, a retrieval cue triggers a pattern completion process that reinstates the encoded event, leading to memory retrieval. This process is thought to be supported by MTL regions, which each serve a functional role (Staresina et al., 2013; Teyler & Rudy, 2007). Specifically, the Binding of Items in Contexts (BIC) model proposes that the perirhinal cortex supports item‐based recognition, while the parahippocampal cortex supports contextual recognition, and the hippocampus is involved in the binding between items and their context, needed for associative memory (Aggleton & Brown, 1999; Diana et al., 2010; Eichenbaum et al., 2007; Ranganath, 2010). This idea is supported by research showing that the perirhinal cortex is associated with familiarity, activity in the parahippocampal cortex relates to recollection, and the hippocampus is involved in both recollection and associative memory (Diana et al., 2007). In contrast to the BIC model, a different theory proposes that familiarity and recollection signals are present throughout the MTL, but that there is a variable non‐linear fMRI sensitivity to memory strength in different brain regions. FMRI might not be able to pick up subtle differences in activity in the hippocampus and perirhinal cortex, when memory strength is low or high, respectively. This would lead to incorrect assumptions about their roles in recollection and familiarity (Squire et al., 2007). We note that the concept of memory strength encompasses more than solely memory confidence ratings. However, here we will only focus on the aspect of subjectively perceived memory confidence.

Yonelinas et al. (2005) examined word recognition using a combined confidence and RK judgement. In this method, participants are asked to give a ‘remember’ response if they can remember specific details of encountering the stimulus at encoding (i.e., associative memory). If they cannot remember specific details (i.e., item memory), they are asked to rate their familiarity on a confidence scale. It is often assumed that the remember responses are made with highest confidence, which equals or exceeds that of familiar items given a high confidence rating. However, participants are instructed that the choice between ‘remember’ and ‘high‐confident familiarity’ responses is not based on confidence, but on recollected contextual details. Nevertheless, Yonelinas et al. (2005) showed that the hippocampus and parahippocampal cortex both showed greater activity for remember responses as compared with high‐confident familiarity responses. The pattern was replicated in later studies which showed greater hippocampus and/or parahippocampal activity in remember or high‐confident old responses, as opposed to lower‐confident responses (Daselaar, Fleck, Dobbins, et al., 2006; Dew et al., 2014; Diana et al., 2010; Hayes et al., 2011; Johnson et al., 2013; Kafkas et al., 2017; Kafkas & Montaldi, 2012; Kirwan et al., 2009; Mendelsohn et al., 2010; Moritz et al., 2006; Mugikura et al., 2010; Risius et al., 2013; Slotnick & Thakral, 2013; Wais, 2011; Wais et al., 2010; Wang, Rogers, et al., 2014). The parahippocampal cortex showed either a positive linear (see Figure 1a) (Kafkas et al., 2017; Woroch et al., 2019) or a recognition threshold pattern (see Figure 1b) (Johnson et al., 2013), and the hippocampus displayed mainly a recognition threshold pattern (Cohn et al., 2009; Daselaar, Fleck, Dobbins, et al., 2006; Johnson et al., 2013; Kafkas et al., 2017; Kafkas & Montaldi, 2012; Kirwan et al., 2009; Montaldi et al., 2006; Slotnick & Thakral, 2013; Wang, Rogers, et al., 2014). Interestingly, the hippocampus can also show a U‐shaped pattern (see Figure 1c) (Hou et al., 2021; Johnson et al., 2013; Kafkas & Montaldi, 2012; Mugikura et al., 2010), suggesting that activity in the hippocampus increases when high‐confident responses are made, irrespective of memory status (Chua et al., 2006; Kim & Cabeza, 2009).

An interesting study specifically focused on the nature of the relationship between the medial temporal lobe regions and memory confidence patterns (Daselaar, Fleck, & Cabeza, 2006). They modelled a recognition threshold‐based, a positive linear, and a negative linear activity pattern. Results showed a triple dissociation: The posterior hippocampus showed a recognition threshold pattern, the posterior parahippocampal cortex showed a positive linear relationship with memory confidence, and the anterior hippocampus showed a negative linear relationship with memory confidence. These results largely mirror the ones discussed above and suggest that subregions of the hippocampus might play different roles in the subjective aspect of memory retrieval. Perhaps dependent on the measurements and the involvement of the anterior hippocampus, either a recognition threshold or a U‐shape pattern can be found in the hippocampus.

Other subregions of the MTL also appear to serve different aspects of subjective memory. Perirhinal cortex activity can be characterized as a positive linear (Kafkas et al., 2017; Woroch et al., 2019), negative linear (Kafkas et al., 2017; Montaldi et al., 2006), inverted U‐shaped (Kafkas & Montaldi, 2012; Mugikura et al., 2010), or U‐shaped pattern (Hou et al., 2021; Wang, Rogers, et al., 2014). In this latter study, they found a threshold‐like response when comparing ‘high‐confident old’ responses to the lower confidence levels and a negative linear response in those lower levels of confidence. This was interpreted as both familiarity‐ and recollection‐related activity in the perirhinal cortex. If the perirhinal cortex is indeed involved in multiple processes during memory retrieval, this could explain the variability in confidence‐related activity patterns reported. The temporopolar area can show a recognition threshold (Johnson et al., 2013), positive linear (Kirwan et al., 2009), or negative linear (Kirwan et al., 2009) pattern. The fusiform gyrus can either show a positive linear (Kafkas et al., 2017), recognition threshold‐like or a U‐shaped pattern (Hutchinson et al., 2015; Johnson et al., 2013) and the rhinal cortex activity decreases (Daselaar, Fleck, & Cabeza, 2006; Daselaar, Fleck, Dobbins, et al., 2006; Kirwan et al., 2009) or increases (Kirwan et al., 2009) linearly with memory confidence. Because these areas are studied less as compared with the hippocampus, it is unclear whether these memory confidence patterns are dependent on task‐specific features.

In summary, most of the studies find effects in the hippocampus, specifically recognition threshold patterns. Given its proposed role in the binding of information, this may be especially important during high‐confident memory retrieval. Fewer studies have found effects in the parahippocampal and perirhinal cortex. The parahippocampal cortex seems to mirror hippocampal patterns in showing mainly a positive linear or recognition threshold effect. On the other hand, the perirhinal cortex seems to show variable patterns, possibly indicating involvement in multiple retrieval‐related processes.

### 3.2 Prefrontal cortex

In addition to the reinstatement of the encoded events through MTL regions, cognitive control is needed for the successful retrieval of information that is relevant for the current task goals. Similar to its role in memory encoding, the VLPFC is important for cognitive control during retrieval as well. When the demand for controlled retrieval increases, so does the activity in the VLPFC (Barredo et al., 2015). The VLPFC assists controlled access to stored representations and supports post‐retrieval operations that resolves competition amongst these representations (Badre & Wagner, 2007; Nyhus & Badre, 2015). It may be that the VLPFC does not play a direct role in memory retrieval (Medvedeva et al., 2019), but that through post‐retrieval monitoring, it plays a more prominent role in subjective memory. Post‐retrieval monitoring demands are increased when there is memory uncertainty and top‐down control is needed to guide memory‐related decision making (Allan et al., 2001; Dulas & Duarte, 2013; Horne et al., 2021; Nyhus & Badre, 2015; Nyhus & Curran, 2010). The DLPFC has also been associated with post‐retrieval processes (Achim & Lepage, 2005; Henson et al., 2000; Rugg, 2004; Rugg et al., 2003) and its activity reflects various decisions that are made prior to making a behavioural response (Dobbins & Han, 2006; Hayama & Rugg, 2009). Interestingly, Chua and Ahmed (2016) reported that excitatory tDCS over the DLPFC improved memory monitoring, while it had no effect on objective memory performance. Just like the DLPFC and VLPFC, retrieval‐related vmPFC activity has also been linked to memory monitoring. It has been proposed that the vmPFC represents the context, events, and responses associated with a memory, functionally interacting with the hippocampus (Euston et al., 2012). Over time, these will generalize and the vmPFC becomes involved in the formation of memory schemas, which can be used to monitor the accuracy of memories (Euston et al., 2012; Hebscher & Gilboa, 2016). This ‘feeling of rightness’ monitoring is essential to avoid confabulations and might have a large influence on subjectively perceived memory confidence (Hebscher & Gilboa, 2016). To summarize, it appears that activity in various PFC regions support the decision‐making process involved in memory.

Memory confidence effects in the VLPFC are mainly reported in the left hemisphere and show various patterns (Cohn et al., 2009; Daselaar, Fleck, & Cabeza, 2006; Daselaar, Fleck, Dobbins, et al., 2006; Hutchinson et al., 2015; Johnson et al., 2013; Kafkas & Montaldi, 2012; Mayes et al., 2019; Montaldi et al., 2006; Yonelinas et al., 2005). Therefore, this PFC region is involved in subjective memory confidence, but its involvement appears inconsistent. Overall, it appears that, within the VLPFC, the pars triangularis and pars opercularis (BA44/45) are more involved in low‐confident memory recognition (Daselaar, Fleck, & Cabeza, 2006; Daselaar, Fleck, Dobbins, et al., 2006; Hutchinson et al., 2015; Johnson et al., 2013; Kim & Cabeza, 2009; Risius et al., 2013; Yonelinas et al., 2005). In the left hemisphere, these areas comprise Broca's area and thus may be specific to top‐down processes related to verbal information. In case there is more uncertainty about the retrieved information, there is an increased need for top‐down post‐retrieval monitoring, reflected in higher VLPFC activation (Nyhus & Badre, 2015). Another language area, the pars orbitalis (BA47), seemed to mainly show sensitivity to high‐confident responses, especially towards old items (Daselaar, Fleck, & Cabeza, 2006; Hutchinson et al., 2015; Johnson et al., 2013; Montaldi et al., 2006). This region is involved in controlled semantic retrieval (Sabb et al., 2007), and thus its involvement in memory confidence may be specific to verbal information as well.

As was the case for the VLPFC, effects in the DLPFC seem to be mostly left‐lateralized and diverse in nature. Confidence‐related activity patterns in the DLPFC mainly show a positive linear pattern (Cohn et al., 2009; Hou et al., 2021; Hutchinson et al., 2015; Johnson et al., 2013; Kafkas & Montaldi, 2012; Mayes et al., 2019), but various other patterns are also reported (Daselaar, Fleck, & Cabeza, 2006; Fleck et al., 2006; Hutchinson et al., 2015; Johnson et al., 2013; Kafkas & Montaldi, 2012; Montaldi et al., 2006). Therefore, it appears that the DLPFC is involved in the subjective aspects of recognition memory, but that the specific relation is not uniform. For instance, Hutchinson et al. (2015) recorded brain activity while participants were retrieving previously learned words. They looked at brain areas showing a positive linear, U‐shape, or inverted U‐shape memory confidence pattern, and showed that all patterns were found in DLPFC regions. Even within a single study, various DLFPC regions were reported to show different patterns, making it unlikely that task‐specific features are the cause for these differences. Instead, it seems that there may be functional subdivisions within the DLPFC, responsible for different aspects of memory retrieval.

In the vmPFC there seems to be a functional dissociation between the left anterior and right posterior regions. The left anterior PFC and orbitofrontal cortex mainly show an increase in activity when memory confidence increases (Chua et al., 2006; Daselaar, Fleck, & Cabeza, 2006; Daselaar, Fleck, Dobbins, et al., 2006; Hou et al., 2021; Hutchinson et al., 2015; Johnson et al., 2013; Mayes et al., 2019; Yonelinas et al., 2005), either in a U‐shape (Hou et al., 2021; Hutchinson et al., 2015; Moritz et al., 2006; Yonelinas et al., 2005), a recognition threshold (Daselaar, Fleck, & Cabeza, 2006; Daselaar, Fleck, Dobbins, et al., 2006; Johnson et al., 2013), or a positive linear pattern (Yonelinas et al., 2005), whereas right‐lateralized activity in the anterior cingulate cortex shows a more variable response to memory confidence, which can be both increased and decreased (Chua et al., 2006; Daselaar, Fleck, & Cabeza, 2006; Daselaar, Fleck, Dobbins, et al., 2006; Fleck et al., 2006; Johnson et al., 2013; Kim & Cabeza, 2009; Moritz et al., 2006; Yonelinas et al., 2005). So, within the vmPFC there appears to be a distinction between the more anterior region, showing a left‐lateralized increase in activity when memory confidence increases, and the more posterior region, showing either an increase or decrease in activity during memory confidence decisions.

Given this variability in activity patterns, it is likely that the contribution of the PFC to memory recognition is through various subprocesses that indirectly influence memory confidence. These are likely related to post‐retrieval control mechanisms or language‐related processes.

### 3.3 Posterior parietal cortex

The influence of the PPC in memory retrieval, might be through attention, as was proposed for its influence during encoding. For instance, the Attention to Memory (AtoM) model predicts that during memory retrieval, the VPC monitors the retrieved information through bottom‐up attention, while the DPC exerts top‐down control over MTL regions (Cabeza et al., 2008; Ciaramelli et al., 2008). High‐confident recognition likely coincides with a rapid retrieval of ample information, capturing bottom‐up attention mediated by the VPC. On the contrary, low‐confident retrieval likely is accompanied by a less efficient initial retrieval attempt, requiring a top‐down controlled memory search mediated by the DPC. Complementary to this, the Integrative Memory model states that the level of processing fluency is monitored regarding an internal criterion (Bastin et al., 2019). When the fluency level exceeds this internal criterion, this can cause a feeling of familiarity. The judgement of whether the fluency of an item is due to familiarity, occurs when recognition decisions are made with DPC‐supported top‐down attention (Bastin et al., 2019). These processes might also influence the way we subjectively perceive the accuracy of a memory. If X amount of retrieved information is needed to make an ‘old’ decision, if more than X amount of information is retrieved, subjectively perceived confidence in the memory will increase.

One of the VPC regions that is consistently linked to memory confidence is the angular gyrus, especially in the left hemisphere. In general, given its association with recollection, it is believed that activity in the left angular gyrus is linked to high confident recognition of old items (Ramanan et al., 2018; Tibon et al., 2019). And indeed, when combined confidence and RK judgements are made, participants show greater left angular gyrus activation during remember responses than during high‐confident familiarity responses (Hou et al., 2021; Johnson et al., 2013; Kafkas & Montaldi, 2012; Yonelinas et al., 2005). Also, in other studies, the left angular gyrus consistently shows higher activation for high‐confident memories (Cohn et al., 2009; Daselaar, Fleck, & Cabeza, 2006; Daselaar, Fleck, Dobbins, et al., 2006; Johnson et al., 2013; Kim & Cabeza, 2009; Mayes et al., 2019; Mendelsohn et al., 2010; Montaldi et al., 2006; Moritz et al., 2006; Wais, 2011; Yonelinas et al., 2005). Although often a positive linear or recollection threshold relationship is assumed, some studies have reported a U‐shaped pattern (Daselaar, Fleck, & Cabeza, 2006; Daselaar, Fleck, Dobbins, et al., 2006; Hou et al., 2021; Hutchinson et al., 2015). This would indicate that the left angular gyrus is not only sensitive to high‐confident recognition, but also to high‐confident novelty detection. However, a TMS study found that inhibiting the angular gyrus only had an influence on confidence for old items (Wynn et al., 2018). Therefore, the influence of the AG on high‐confident recognition might be larger than its influence on high‐confident novelty detection. Other VPC areas, including the inferior parietal lobe, supramarginal gyrus and the temporoparietal junction, have been linked to memory confidence in a similar way. As with the angular gyrus, these VPC regions appear to show greater activation during high‐confident memory retrieval (Cohn et al., 2009; Daselaar, Fleck, & Cabeza, 2006; Daselaar, Fleck, Dobbins, et al., 2006; Hayes et al., 2011; Hutchinson et al., 2015; Johnson et al., 2013; Leiker & Johnson, 2015; Yonelinas et al., 2005). Taken together, the VPC seems to be specifically sensitive to high‐confident memory retrieval, concurring with its proposed link to recollection (Cabeza et al., 2008, 2011).

The DPC regions of main interest here are the superior parietal lobe, intraparietal sulcus, and precuneus. Just as with the VPC regions, activity in DPC regions has been shown to increase with memory confidence (Cohn et al., 2009; Daselaar, Fleck, Dobbins, et al., 2006; Hou et al., 2021; Hutchinson et al., 2015; Johnson et al., 2013; Mayes et al., 2019; Montaldi et al., 2006; Yonelinas et al., 2005). However, the DPC regions do not seem to show increased activity in remember or high‐confident old responses (Cohn et al., 2009; Daselaar, Fleck, & Cabeza, 2006; Hou et al., 2021; Hutchinson et al., 2015; Johnson et al., 2013; Kim & Cabeza, 2009; Mayes et al., 2019; Yonelinas et al., 2005) and activity can show a negative linear pattern (Daselaar, Fleck, Dobbins, et al., 2006; Fleck et al., 2006; Woroch et al., 2019), indicating that these regions might be of greater importance when there is memory uncertainty. This interpretation is supported by reports of an inverted U‐shaped relation between DPC regions and memory confidence (Hutchinson et al., 2015; Moritz et al., 2006). DPC activity has been linked to familiarity before (Cabeza et al., 2011; Ciaramelli et al., 2008), but these findings suggest that a more accurate interpretation of the activity patterns might be memory uncertainty. When unsure about the memory status of an item, DPC regions might become involved in a frontoparietal network to support decision making, as proposed by the Mnemonic Accumulator hypothesis (Wagner et al., 2005). According to this hypothesis, the PPC integrates information from other areas (e.g., MTL and sensory areas), contributing to memory decision making processes, which determines memory confidence.

To summarize, the retrieval‐related effects reported in the PPC are in concordance with the literature and specifically the assumptions put forward by the AtoM model. The VPC seems to be involved in the bottom‐up attention capture by high‐confident memories, while the DPC activity patterns indicate involvement in top‐down controlled processes during low‐confident memories. In addition, complementary processes seem to occur during novelty processes in these two brain areas.

### 3.4 Overall effects on memory confidence

**FIGURE 3:**
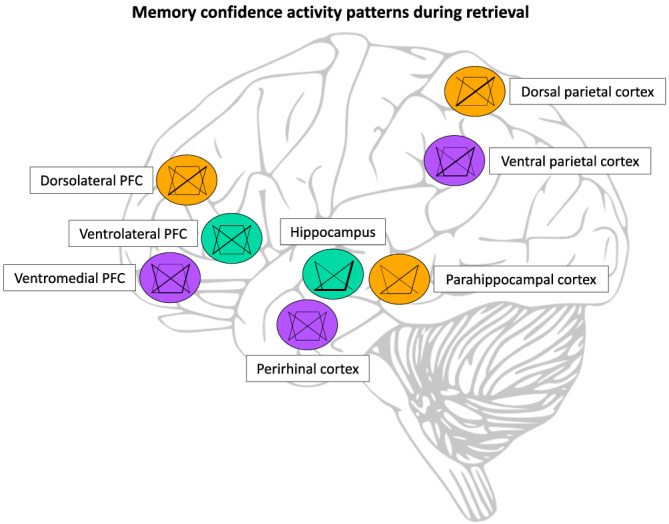
Summary of the retrieval‐related activity patterns in relation to confidence levels in the relevant brain regions. Line thickness is scaled according to the number of studies supporting the pattern

The retrieval‐related confidence activity patterns in the MTL, PFC, and PPC indicate their possible role in memory confidence during retrieval (see Figure 3 and Table 2). The sharp increase in hippocampal activity in high‐confident old responses seems to indicate the hippocampus is mainly involved when there is little memory uncertainty. The activity appears threshold‐based and only when a certain amount of memory evidence is reached, activity increases considerably. This concurs with the notion that the hippocampus is involved in recollection (Eichenbaum et al., 2007; Ranganath, 2010). The hippocampus might be essential in high‐confident responses that are mainly based on the binding of contextual information (Hayes et al., 2011; Kafkas et al., 2017; Leiker & Johnson, 2015; Mayes et al., 2019). Likewise, the activity pattern in the parahippocampal cortex seems to primarily increase with memory confidence, either positively linear or in a recognition threshold manner. During memory retrieval, the parahippocampal cortex is important for representations of the encoding context of remembered items (Eichenbaum et al., 2007). This indicates that the stronger the representation of the encoding context, the higher the memory confidence ratings. Where the hippocampus has been deemed important for recollection, the parahippocampal cortex assists recollection through retrieval of contextual information, and the perirhinal cortex has been linked to familiarity (Eichenbaum et al., 2007; Ranganath, 2010). Familiarity is not assumed to be threshold‐based, and it has been proposed that fMRI measurements have difficulty dissociating between moderate and strong memories in the perirhinal cortex (Squire et al., 2007). Therefore, it is unexpected that U‐shape patterns are found in the perirhinal cortex. In these cases, the perirhinal cortex might be important for high‐confident responses that are based mainly on item information (Diana et al., 2010), which in most studies would be classified as familiarity‐based responses. Given the reports of U‐shaped patterns in both the perirhinal cortex and hippocampus, it appears that these brain regions can also be involved in high‐confident memory rejection, or novelty detection, perhaps in dedicated subregions (Axmacher et al., 2010; Bogacz et al., 2001; Jeewajee et al., 2008; VanElzakker et al., 2008).

Interestingly, none of the three predefined PFC regions showed a consistent pattern of confidence related brain activity, indicating no direct uniform role in memory confidence. A reason for this might be the post‐retrieval monitoring supported by PFC regions (Achim & Lepage, 2005; Henson et al., 2000; Nyhus & Badre, 2015; Rugg et al., 2003). If more activity in these brain regions means more post‐retrieval monitoring, this could be independent from confidence ratings. More monitoring does not necessarily mean more successful evidence accumulation or higher memory accuracy. It just means that attempts are made to reduce the ambiguity in the initial retrieved information, but this is not necessarily successful (Henson et al., 2000). This would explain the inconsistency in confidence‐related patterns: When post‐retrieval monitoring was successful, memory confidence will be higher, when it is not, memory confidence will be lower. In addition, there also appears to be functional differentiation in the PFC subregions. Some subregions might play a specific role in post‐retrieval monitoring, language processing, or both. Given that most memory task had a verbal element, it is difficult to say if the reported effects would be the same if non‐verbal tasks were used. Nevertheless, we can conclude that the DLPFC, VLPFC, and vmPFC play no direct uniform role in memory confidence.

The VPC and DPC showed complementary patterns, with the VPC mainly being involved in high‐confident memory, while the DPC is mainly involved in the lower levels of memory confidence (Hayes et al., 2011). These results match those proposed by the AtoM model (Ciaramelli et al., 2008). They hypothesized that high‐confident memories would automatically capture attention in a bottom‐up fashion. On the other hand, it is thought that when confidence is lower, there is more need for memory search attempts and post‐retrieval monitoring processes, which require top‐down attention. While the AtoM model mainly focuses on the role of confidence in old items, the findings shown here suggest that this may also translate to novel items. Mechanisms for novelty detection may be similar, when there is uncertainty regarding the novelty, top‐down attention is needed to guide a memory‐search, checking if the information is indeed new. A strong novelty signal would capture bottom‐up attention in a similar manner as a strong memory signal. This would support the notion that the PPC is involved in the evaluation and decision making process, irrespective of memory status of the information (Rutishauser et al., 2018).

## 4 CONCLUSION

There is a wealth of literature on brain activity related to memory processes, which has identified the involvement of the MTL, PFC, and PPC. Most of these studies give insight on the processes involved in objective memory performance, in what makes us remember and forget, but how we subjectively perceive memories is often overlooked. Researchers incorporate confidence ratings in their measurements, but they are not always used to their full potential nor analysed consistently. Here, we reviewed the neuroscientific literature on subjectively perceived memory confidence and described specific brain activity patterns.

Activity in the hippocampus and parahippocampal cortex seems to mainly increase with memory confidence. In both the encoding and retrieval phase, activity mainly shows a positive linear or a recognition threshold pattern. This pattern can be explained by processes involved in both the binding of elements during encoding and the retrieval of this associative information during retrieval. When more elements are associated with a single memory event, the confidence in this event increases. Particularly when there is a rich and detailed memory, the highest level of confidence in the accuracy of the retrieved information can be attained. On the other hand, the perirhinal cortex does not show a recognition threshold pattern, in neither encoding nor retrieval. The positive linear pattern that this brain area shows during encoding is most likely related to its role in the encoding of item information. However, during retrieval, various perirhinal cortex activity patterns are observed, which does not directly match with its proposed role in familiarity processes. It seems that during retrieval, the perirhinal cortex can be involved in multiple processes involved in both memory retrieval and novelty detection.

Memory confidence related brain activity patterns in the PFC regions appear largely inconsistent during both encoding and retrieval. Multiple patterns are found across and within studies, which indicates different functional roles for various subregions within the VLPFC, DLPFC, and vmPFC. One of the roles is likely related to control mechanisms involved in attention during encoding and post‐retrieval monitoring during retrieval. The need for and effect of these control mechanisms may be variable and dependent on task‐related characteristics, like demand for top‐down control. In addition, there appears to be a lateralisation in the effects found in the VLPFC and DLPFC. The VLPFC mainly shows a lateralisation based upon stimulus material, with the left VLPFC mainly involved in the memory processes regarding verbal information. In the DLPFC, left lateralized encoding‐related activity seems to be beneficial to subsequent memory confidence, while the opposite holds for right‐lateralized encoding‐related activity. However, these lateralisation effects may be moderated by task‐specific variables, like the difference between item and source memory tasks. During retrieval, various subregions in the DLPFC and vmPFC seem to present with different confidence‐related activity patterns and thus may serve specific subfunctions. The nature of these subfunctions likely relate to post‐retrieval monitoring and language processes.

FMRI results regarding encoding‐related PPC activity point towards mixed patterns in both the VPC and DPC. Both regions show positive and negative linear patterns, which show some contradiction with their proposed roles in attentional processes. Moreover, NIBS studies show no clear effects of brain stimulation on the effects on subsequent memory confidence. Together, these studies do not support the idea that during memory encoding the VPC and DPC play a direct or causal role in subsequent memory confidence. On the contrary, retrieval‐related confidence patterns in the VPC and DPC concur with the patterns proposed by the AtoM model. VPC‐mediated bottom‐up processes appear to be involved in high‐confident recognition, while DPC‐mediated top‐down processes are likely involved in low‐confident recognition. In addition, these patterns appear to be related to novelty processes as well, with the VPC showing increased activity for high‐confident novelty detection and the DPC showing increased activity for low‐confident novelty detection.

Here, we focused solely on explicit reports of memory confidence by reviewing studies that have used overt responses participants made regarding their confidence. We only included studies concerning healthy adults, and therefore, we have no reason to assume their explicit memory confidence would be impaired. However, amnesic patients can show impairments in explicit memory measures, while implicit memory can be intact (Chun, 2005; Golby et al., 2005; Langer, 2021). This same pattern has been found in patients with MTL lesions, who show impairments in detecting regularities, or statistical learning, when assessed explicitly (Schapiro et al., 2014), but show no impairments on implicit measures (Rungratsameetaweemana et al., 2019). When investigating memory confidence in patient groups that show impairments in explicit memory, learning, or decision making, reaction times could be used as a proxy for memory confidence, in addition to confidence ratings. In healthy participants there is an U‐shaped relationship between explicitly reported memory confidence and the reaction time on ‘old/new’ responses, with RTs being the shortest for high‐confidence responses and the highest for low‐confidence responses (Starns, 2021; Weidemann & Kahana, 2016; Yonelinas et al., 2005). In patient groups, explicit memory confidence may be impaired, but reaction times may still be used as a measure of implicit memory confidence.

To summarize, the brain mechanisms involved in objective memory encoding and retrieval also contribute to subjective memory. Understanding the functional organization of the brain that underlies memory confidence is not only important for understanding more subtle brain‐behaviour relations that may be clinically relevant, but also contributes to understanding memory‐guided decision making.

## CONFLICT OF INTEREST

The authors declare that there is no conflict of interest.

5

### PEER REVIEW

The peer review history for this article is available at https://publons.com/publon/10.1111/ejn.15649.

## Data Availability

Data sharing is not applicable to this article as no new data were created or analyzed in this study.

## References

[ejn15649-bib-0001] Achim, A. M. , & Lepage, M. (2005). Dorsolateral prefrontal cortex involvement in memory post‐retrieval monitoring revealed in both item and associative recognition tests. NeuroImage, 24(4), 1113–1121. 10.1016/j.neuroimage.2004.10.036 15670688

[ejn15649-bib-0002] Addante, R. J. , Ranganath, C. , & Yonelinas, A. P. (2012). Examining ERP correlates of recognition memory: Evidence of accurate source recognition without recollection. NeuroImage, 62(1), 439–450. 10.1016/j.neuroimage.2012.04.031 22548808 PMC3381051

[ejn15649-bib-0003] Aggleton, J. P. , & Brown, M. W. (1999). Episodic memory, amnesia, and the hippocampal‐anterior thalamic axis. The Behavioral and Brain Sciences, 22(3), 444–489. 10.1017/S0140525X99002034 11301518

[ejn15649-bib-0004] Aggleton, J. P. , & Brown, M. W. (2006). Interleaving brain systems for episodic and recognition memory. Trends in Cognitive Sciences, 10, 455–463. 10.1016/j.tics.2006.08.003 16935547

[ejn15649-bib-0005] Alekseichuk, I. , Turi, Z. , Veit, S. , & Paulus, W. (2020). Model‐driven neuromodulation of the right posterior region promotes encoding of long‐term memories. Brain Stimulation, 13, 474–483. 10.1016/j.brs.2019.12.019 31882373

[ejn15649-bib-0006] Allan, K. , Wolf, H. A. , Rosenthal, C. R. , & Rugg, M. D. (2001). The effect of retrieval cues on post‐retrieval monitoring in episodic memory: An electrophysiological study. Cognitive Brain Research, 12, 289–299. 10.1016/S0926-6410(01)00061-1 11587897

[ejn15649-bib-0007] Awipi, T. , & Davachi, L. (2008). Content‐specific source encoding in the human medial temporal lobe. Journal of Experimental Psychology. Learning, Memory, and Cognition, 34(4), 769–779. 10.1037/0278-7393.34.4.769 18605867 PMC2938959

[ejn15649-bib-0008] Axmacher, N. , Cohen, M. X. , Fell, J. , Haupt, S. , Dümpelmann, M. , Elger, C. E. , Schlaepfer, T. E. , Lenartz, D. , Sturm, V. , & Ranganath, C. (2010). Intracranial EEG correlates of expectancy and memory formation in the human hippocampus and nucleus accumbens. Neuron, 65(4), 541–549. 10.1016/j.neuron.2010.02.006 20188658

[ejn15649-bib-0009] Badre, D. , & Wagner, A. D. (2007). Left ventrolateral prefrontal cortex and the cognitive control of memory. Neuropsychologia, 45(13), 2883–2901. 10.1016/j.neuropsychologia.2007.06.015 17675110

[ejn15649-bib-0010] Barredo, J. , Oztekin, I. , & Badre, D. (2015). Ventral fronto‐temporal pathway supporting cognitive control of episodic memory retrieval. Cerebral Cortex, 25(4), 1004–1019. 10.1093/cercor/bht291 24177990 PMC4366615

[ejn15649-bib-0011] Bastin, C. , Besson, G. , Simon, J. , Delhaye, E. , Geurten, M. , Willems, S. , & Salmon, E. (2019). An integrative memory model of recollection and familiarity to understand memory deficits. The Behavioral and Brain Sciences, 42, e281. 10.1017/S0140525X19000621 30719958

[ejn15649-bib-0012] Benoit, R. G. , & Schacter, D. L. (2015). Specifying the core network supporting episodic simulation and episodic memory by activation likelihood estimation. Neuropsychologia, 75, 450–457. 10.1016/j.neuropsychologia.2015.06.034 26142352 PMC4546530

[ejn15649-bib-0013] Bjekic, J. , Colic, M. V. , Zivanovic, M. , Milanovic, S. D. , & Filipovic, S. R. (2019). Transcranial direct current stimulation (tDCS) over parietal cortex improves associative memory. Neurobiology of Learning and Memory, 157, 114–120. 10.1016/j.nlm.2018.12.007 30553021

[ejn15649-bib-0014] Blumenfeld, R. S. , Parks, C. M. , Yonelinas, A. P. , & Ranganath, C. (2011). Putting the pieces together: The role of dorsolateral prefrontal cortex in relational memory encoding. Journal of Cognitive Neuroscience, 23(1), 257–265. 10.1162/jocn.2010.21459 20146616 PMC3970078

[ejn15649-bib-0015] Blumenfeld, R. S. , & Ranganath, C. (2007). Prefrontal cortex and long‐term memory encoding: An integrative review of findings from neuropsychology and neuroimaging. The Neuroscientist, 13(3), 280–291. 10.1177/1073858407299290 17519370

[ejn15649-bib-0016] Bogacz, R. , Brown, M. W. , & Giraud‐Carrier, C. (2001). Model of familiarity discrimination in the perirhinal cortex. Journal of Computational Neuroscience, 10, 5–23. 10.1023/A:1008925909305 11316340

[ejn15649-bib-0017] Brewer, N. , & Burke, A. (2002). Effects of testimonial inconsistencies and eyewitness confidence on mock‐juror judgments. Law and Human Behavior, 26, 353–364. 10.1023/A:1015380522722 12061623

[ejn15649-bib-0018] Brod, G. , & Shing, Y. L. (2018). Specifying the role of the ventromedial prefrontal cortex in memory formation. Neuropsychologia, 111, 8–15. 10.1016/j.neuropsychologia.2018.01.005 29317324

[ejn15649-bib-0019] Brown, M. W. , & Aggleton, J. P. (2001). Recognition memory: What are the roles of the perirhinal cortex and hippocampus? Nature Reviews Neuroscience, 2(1), 51–61. 10.1038/35049064 11253359

[ejn15649-bib-0020] Cabeza, R. , Ciaramelli, E. , Olson, I. R. , & Moscovitch, M. (2008). The parietal cortex and episodic memory: An attentional account. Nature Reviews. Neuroscience, 9(8), 613–625. 10.1038/nrn2459 18641668 PMC2692883

[ejn15649-bib-0021] Cabeza, R. , Mazuz, Y. S. , Stokes, J. , Kragel, J. E. , Woldorff, M. G. , Ciaramelli, E. , Olson, I. R. , & Moscovitch, M. (2011). Overlapping parietal activity in memory and perception: Evidence for the attention to memory model. Journal of Cognitive Neuroscience, 23(11), 3209–3217. 10.1162/jocn_a_00065 21568633 PMC3518433

[ejn15649-bib-0022] Carr, V. A. , Viskontas, I. V. , Engel, S. A. , & Knowlton, B. J. (2010). Neural activity in the hippocampus and perirhinal cortex during encoding is associated with the durability of episodic memory. Journal of Cognitive Neuroscience, 22(11), 2652–2662. 10.1162/jocn.2009.21381 19925190

[ejn15649-bib-0023] Chua, E. F. , & Ahmed, R. (2016). Electrical stimulation of the dorsolateral prefrontal cortex improves memory monitoring. Neuropsychologia, 85, 74–79. 10.1016/j.neuropsychologia.2016.03.008 26970142 PMC4853246

[ejn15649-bib-0024] Chua, E. F. , Hannula, D. E. , & Ranganath, C. (2012). Distinguishing highly confident accurate and inaccurate memory: Insights about relevant and irrelevant influences on memory confidence. Memory, 20(1), 48–62. 10.1080/09658211.2011.633919 22171810 PMC3269835

[ejn15649-bib-0025] Chua, E. F. , Schacter, D. L. , Rand‐Giovannetti, E. , & Sperling, R. A. (2006). Understanding metamemory: Neural correlates of the cognitive process and subjective level of confidence in recognition memory. NeuroImage, 29(4), 1150–1160. 10.1016/j.neuroimage.2005.09.058 16303318

[ejn15649-bib-0026] Chun, M. M. (2005). Drug‐induced amnesia impairs implicit relational memory. Trends in Cognitive Sciences, 9(8), 355–357. 10.1016/j.tics.2005.06.015 16006177

[ejn15649-bib-0027] Ciaramelli, E. , Grady, C. L. , & Moscovitch, M. (2008). Top‐down and bottom‐up attention to memory: A hypothesis (AtoM) on the role of the posterior parietal cortex in memory retrieval. Neuropsychologia, 46(7), 1828–1851. 10.1016/j.neuropsychologia.2008.03.022 18471837

[ejn15649-bib-0028] Cohn, M. , Moscovitch, M. , Lahat, A. , & McAndrews, M. P. (2009). Recollection versus strength as the primary determinant of hippocampal engagement at retrieval. Proceedings of the National Academy of Sciences of the United States of America, 106(52), 22451–22455. 10.1073/pnas.0908651106 20007783 PMC2799749

[ejn15649-bib-0029] Cramer, R. J. , Brodsky, S. L. , & DeCoster, J. (2009). Expert witness confidence and juror personality: Their impact on credibility and persuasion in the courtroom. Journal of the American Academy of Psychiatry and the Law Online, 37, 63–74. 19297636

[ejn15649-bib-0030] Daselaar, S. M. , Fleck, M. S. , & Cabeza, R. (2006). Triple dissociation in the medial temporal lobes: Recollection, familiarity, and novelty. Journal of Neurophysiology, 96(4), 1902–1911. 10.1152/jn.01029.2005 16738210

[ejn15649-bib-0031] Daselaar, S. M. , Fleck, M. S. , Dobbins, I. G. , Madden, D. J. , & Cabeza, R. (2006). Effects of healthy aging on hippocampal and rhinal memory functions: An event‐related fMRI study. Cerebral Cortex, 16(12), 1771–1782. 10.1093/cercor/bhj112 16421332 PMC1810232

[ejn15649-bib-0032] Daselaar, S. M. , Prince, S. E. , & Cabeza, R. (2004). When less means more: Deactivations during encoding that predict subsequent memory. NeuroImage, 23(3), 921–927. 10.1016/j.neuroimage.2004.07.031 15528092

[ejn15649-bib-0033] Daselaar, S. M. , Prince, S. E. , Dennis, N. A. , Hayes, S. M. , Kim, H. , & Cabeza, R. (2009). Posterior midline and ventral parietal activity is associated with retrieval success and encoding failure. Frontiers in Human Neuroscience, 3, 13. 10.3389/neuro.09.013.2009 19680466 PMC2726033

[ejn15649-bib-0034] Demeter, E. , Mirdamadi, J. L. , Meehan, S. K. , & Taylor, S. F. (2016). Short theta burst stimulation to left frontal cortex prior to encoding enhances subsequent recognition memory. Cognitive, Affective, & Behavioral Neuroscience, 16(4), 724–735. 10.3758/s13415-016-0426-3 PMC4955696 27098772

[ejn15649-bib-0035] DeSoto, K. A. , & Roediger, H. L. (2014). Positive and negative correlations between confidence and accuracy for the same events in recognition of categorized lists. Psychological Science, 25(3), 781–788. 10.1177/0956797613516149 24452605

[ejn15649-bib-0036] Dew, I. T. , Ritchey, M. , LaBar, K. S. , & Cabeza, R. (2014). Prior perceptual processing enhances the effect of emotional arousal on the neural correlates of memory retrieval. Neurobiology of Learning and Memory, 112, 104–113. 10.1016/j.nlm.2013.12.012 24380867 PMC4051848

[ejn15649-bib-0037] Diana, R. A. , Yonelinas, A. P. , & Ranganath, C. (2007). Imaging recollection and familiarity in the medial temporal lobe: A three‐component model. Trends in Cognitive Sciences, 11(9), 379–386. 10.1016/j.tics.2007.08.001 17707683

[ejn15649-bib-0038] Diana, R. A. , Yonelinas, A. P. , & Ranganath, C. (2010). Medial temporal lobe activity during source retrieval reflects information type, not memory strength. Journal of Cognitive Neuroscience, 22(8), 1808–1818. 10.1162/jocn.2009.21335 19702458 PMC2862119

[ejn15649-bib-0039] Dobbins, I. G. , & Han, S. (2006). Isolating rule‐ versus evidence‐based prefrontal activity during episodic and lexical discrimination: A functional magnetic resonance imaging investigation of detection theory distinctions. Cerebral Cortex, 16(11), 1614–1622. 10.1093/cercor/bhj098 16400153

[ejn15649-bib-0040] Dubravac, M. , & Meier, B. (2021). Stimulating the parietal cortex by transcranial direct current stimulation (tDCS): No effects on attention and memory. AIMS Neurosci, 8(1), 33–46. 10.3934/Neuroscience.2021002 33490371 PMC7815482

[ejn15649-bib-0041] Dulas, M. R. , & Duarte, A. (2013). The influence of directed attention at encoding on source memory retrieval in the young and old: An ERP study. Brain Research, 1500, 55–71. 10.1016/j.brainres.2013.01.018 23348376

[ejn15649-bib-0042] Eichenbaum, H. , Sauvage, M. , Fortin, N. , Komorowski, R. , & Lipton, P. (2012). Towards a functional organization of episodic memory in the medial temporal lobe. Neuroscience and Biobehavioral Reviews, 36(7), 1597–1608. 10.1016/j.neubiorev.2011.07.006 21810443 PMC3227798

[ejn15649-bib-0043] Eichenbaum, H. , Yonelinas, A. P. , & Ranganath, C. (2007). The medial temporal lobe and recognition memory. Annual Review of Neuroscience, 30, 123–152. 10.1146/annurev.neuro.30.051606.094328 PMC2064941 17417939

[ejn15649-bib-0044] Euston, D. R. , Gruber, A. J. , & McNaughton, B. L. (2012). The role of medial prefrontal cortex in memory and decision making. Neuron, 76(6), 1057–1070. 10.1016/j.neuron.2012.12.002 23259943 PMC3562704

[ejn15649-bib-0045] Fleck, M. S. , Daselaar, S. M. , Dobbins, I. G. , & Cabeza, R. (2006). Role of prefrontal and anterior cingulate regions in decision‐making processes shared by memory and nonmemory tasks. Cerebral Cortex, 16(11), 1623–1630. 10.1093/cercor/bhj097 16400154

[ejn15649-bib-0046] Floel, A. , Poeppel, D. , Buffalo, E. A. , Braun, A. , Wu, C. W. , Seo, H. J. , Stefan, K. , Knecht, S. , & Cohen, L. G. (2004). Prefrontal cortex asymmetry for memory encoding of words and abstract shapes. Cerebral Cortex, 14, 404–409. 10.1093/cercor/bhh002 15028644

[ejn15649-bib-0047] Golby, A. , Silverberg, G. , Race, E. , Gabrieli, S. , O'Shea, J. , Knierim, K. , Stebbins, G. , & Gabrieli, J. (2005). Memory encoding in Alzheimer's disease: An fMRI study of explicit and implicit memory. Brain, 128(4), 773–787. 10.1093/brain/awh400 15705615

[ejn15649-bib-0048] Hales, J. B. , & Brewer, J. B. (2011). The timing of associative memory formation: Frontal lobe and anterior medial temporal lobe activity at associative binding predicts memory. Journal of Neurophysiology, 105(4), 1454–1463. 10.1152/jn.00902.2010 21248058 PMC3075291

[ejn15649-bib-0049] Hales, J. B. , & Brewer, J. B. (2013). Parietal and frontal contributions to episodic encoding of location. Behavioural Brain Research, 243, 16–20. 10.1016/j.bbr.2012.12.048 23295390 PMC3593964

[ejn15649-bib-0050] Hayama, H. R. , & Rugg, M. D. (2009). Right dorsolateral prefrontal cortex is engaged during post‐retrieval processing of both episodic and semantic information. Neuropsychologia, 47(12), 2409–2416. 10.1016/j.neuropsychologia.2009.04.010 19383503 PMC2712584

[ejn15649-bib-0051] Hayes, S. M. , Buchler, N. , Stokes, J. , Kragel, J. , & Cabeza, R. (2011). Neural correlates of confidence during item recognition and source memory retrieval: Evidence for both dual‐process and strength memory theories. Journal of Cognitive Neuroscience, 23(12), 3959–3971. 10.1162/jocn_a_00086 21736454 PMC3521503

[ejn15649-bib-0052] Hebscher, M. , & Gilboa, A. (2016). A boost of confidence: The role of the ventromedial prefrontal cortex in memory, decision‐making, and schemas. Neuropsychologia, 90, 46–58. 10.1016/j.neuropsychologia.2016.05.003 27150705

[ejn15649-bib-0053] Henke, K. , Buck, A. , Weber, B. , & Wieser, H. G. (1997). Human hippocampus establishes associations in memory. Hippocampus, 7(3), 249–256. 10.1002/(SICI)1098-1063(1997)7:3<249::AID-HIPO1>3.0.CO;2-G 9228523

[ejn15649-bib-0054] Henson, R. , Rugg, M. , Shallice, T. , & Dolan, R. J. (2000). Confidence in recognition memory for words: Dissociating right prefrontal roles in episodic retrieval. Journal of Cognitive Neuroscience, 12(6), 913–923. 10.1162/08989290051137468 11177413

[ejn15649-bib-0055] Hopkins, R. O. , Kesner, R. P. , & Goldstein, M. (1995). Memory for novel and familiar spatial and linguistic temporal distance information in hypoxic subjects. Journal of the International Neuropsychological Society, 1(5), 454–468. 10.1017/S1355617700000552 9375231

[ejn15649-bib-0056] Horne, E. D. , Chastelaine, M. , & Rugg, M. D. (2021). Neural correlates of post‐retrieval monitoring in older adults are preserved under divided attention, but are decoupled from memory performance. Neurobiology of Aging, 97, 106–119. 10.1016/j.neurobiolaging.2020.10.010 33190122 PMC7736156

[ejn15649-bib-0057] Hou, M. , Wang, T. H. , & Rugg, M. D. (2021). The effects of age on neural correlates of recognition memory: An fMRI study. Brain and Cognition, 153, 105785. 10.1016/j.bandc.2021.105785 34419811 PMC8429125

[ejn15649-bib-0058] Hoven, M. , Lebreton, M. , Engelmann, J. B. , Denys, D. , Luigjes, J. , & Holst, R. J. (2019). Abnormalities of confidence in psychiatry: An overview and future perspectives. Translational Psychiatry, 9, 1–18. 10.1038/s41398-019-0602-7 31636252 PMC6803712

[ejn15649-bib-0059] Hutchinson, J. B. , Uncapher, M. R. , & Wagner, A. D. (2015). Increased functional connectivity between dorsal posterior parietal and ventral occipitotemporal cortex during uncertain memory decisions. Neurobiology of Learning and Memory, 117, 71–83. 10.1016/j.nlm.2014.04.015 24825621 PMC4226743

[ejn15649-bib-0060] Jackson, O. 3rd , & Schacter, D. L. (2004). Encoding activity in anterior medial temporal lobe supports subsequent associative recognition. NeuroImage, 21(1), 456–462. 10.1016/j.neuroimage.2003.09.050 14741683

[ejn15649-bib-0061] Jacobson, L. , Goren, N. , Lavidor, M. , & Levy, D. A. (2012). Oppositional transcranial direct current stimulation (tDCS) of parietal substrates of attention during encoding modulates episodic memory. Brain Research, 1439, 66–72. 10.1016/j.brainres.2011.12.036 22265704

[ejn15649-bib-0062] Jeewajee, A. , Lever, C. , Burton, S. , O'keefe, J. , & Burgess, N. (2008). Environmental novelty is signaled by reduction of the hippocampal theta frequency. Hippocampus, 18(4), 340–348. 10.1002/hipo.20394 18081172 PMC2678674

[ejn15649-bib-0063] Jenkins, L. J. , & Ranganath, C. (2010). Prefrontal and medial temporal lobe activity at encoding predicts temporal context memory. The Journal of Neuroscience, 30(46), 15558–15565. 10.1523/JNEUROSCI.1337-10.2010 21084610 PMC3842495

[ejn15649-bib-0064] Johnson, J. D. , Suzuki, M. , & Rugg, M. D. (2013). Recollection, familiarity, and content‐sensitivity in lateral parietal cortex: A high‐resolution fMRI study. Frontiers in Human Neuroscience, 7, 219. 10.3389/fnhum.2013.00219 23734122 PMC3661949

[ejn15649-bib-0065] Kafkas, A. , Migo, E. M. , Morris, R. G. , Kopelman, M. D. , Montaldi, D. , & Mayes, A. R. (2017). Material specificity drives medial temporal lobe familiarity but not hippocampal recollection. Hippocampus, 27, 194–209. 10.1002/hipo.22683 27859925 PMC5299537

[ejn15649-bib-0066] Kafkas, A. , & Montaldi, D. (2012). Familiarity and recollection produce distinct eye movement, pupil and medial temporal lobe responses when memory strength is matched. Neuropsychologia, 50(13), 3080–3093. 10.1016/j.neuropsychologia.2012.08.001 22902538

[ejn15649-bib-0067] Kahn, I. , Pascual‐Leone, A. , Theoret, H. , Fregni, F. , Clark, D. , & Wagner, A. D. (2005). Transient disruption of ventrolateral prefrontal cortex during verbal encoding affects subsequent memory performance. Journal of Neurophysiology, 94(1), 688–698. 10.1152/jn.01335.2004 15758048

[ejn15649-bib-0068] Kim, H. , & Cabeza, R. (2009). Common and specific brain regions in high‐ versus low‐confidence recognition memory. Brain Research, 1282, 103–113. 10.1016/j.brainres.2009.05.080 19501072 PMC2709704

[ejn15649-bib-0069] Kirwan, C. B. , Shrager, Y. , & Squire, L. R. (2009). Medial temporal lobe activity can distinguish between old and new stimuli independently of overt behavioral choice. Proceedings of the National Academy of Sciences of the United States of America, 106(34), 14617–14621. 10.1073/pnas.0907624106 19706549 PMC2732796

[ejn15649-bib-0070] Kirwan, C. B. , & Stark, C. E. (2004). Medial temporal lobe activation during encoding and retrieval of novel face‐name pairs. Hippocampus, 14(7), 919–930. 10.1002/hipo.20014 15382260 PMC2704554

[ejn15649-bib-0071] Kirwan, C. B. , Wixted, J. T. , & Squire, L. R. (2008). Activity in the medial temporal lobe predicts memory strength, whereas activity in the prefrontal cortex predicts recollection. The Journal of Neuroscience, 28(42), 10541–10548. 10.1523/JNEUROSCI.3456-08.2008 18923030 PMC2590932

[ejn15649-bib-0072] Koen, J. D. , Thakral, P. P. , & Rugg, M. D. (2018). Transcranial magnetic stimulation of the left angular gyrus during encoding does not impair associative memory performance. Cognitive Neuroscience, 9(3–4), 127–138. 10.1080/17588928.2018.1484723 29870300 PMC6185791

[ejn15649-bib-0073] Kohler, S. , Paus, T. , Buckner, R. L. , & Milner, B. (2004). Effects of left inferior prefrontal stimulation on episodic memory formation: A two‐stage fMRI‐rTMS study. Journal of Cognitive Neuroscience, 16, 178–188. 10.1162/089892904322984490 15068590

[ejn15649-bib-0074] Langer, K. G. (2021). The history of amnesia—A review. Current Neurology and Neuroscience Reports, 21(8), 1–7. 10.1007/s11910-021-01126-x 34110519

[ejn15649-bib-0075] Leiker, E. K. , & Johnson, J. D. (2015). Pattern reactivation co‐varies with activity in the core recollection network during source memory. Neuropsychologia, 75, 88–98. 10.1016/j.neuropsychologia.2015.05.021 26004057

[ejn15649-bib-0076] Libby, L. A. , Ekstrom, A. D. , Ragland, J. D. , & Ranganath, C. (2012). Differential connectivity of perirhinal and parahippocampal cortices within human hippocampal subregions revealed by high‐resolution functional imaging. Journal of Neuroscience, 32(19), 6550–6560. 10.1523/JNEUROSCI.3711-11.2012 22573677 PMC3374643

[ejn15649-bib-0077] Loftus, E. F. , & Pickrell, J. E. (1995). The formation of false memories. SLACK Incorporated Thorofare, NJ, 25(12), 720–725. 10.3928/0048-5713-19951201-07

[ejn15649-bib-0078] Maass, A. , Berron, D. , Libby, L. A. , Ranganath, C. , & Düzel, E. (2015). Functional subregions of the human entorhinal cortex. eLife, 4, e06426. 10.7554/eLife.06426 PMC4458841 26052749

[ejn15649-bib-0079] Mayes, A. R. , Montaldi, D. , Roper, A. , Migo, E. M. , Gholipour, T. , & Kafkas, A. (2019). Amount, not strength of recollection, drives hippocampal activity: A problem for apparent word familiarity‐related hippocampal activation. Hippocampus, 29(1), 46–59. 10.1002/hipo.23031 30411437 PMC6492455

[ejn15649-bib-0080] Medvedeva, A. , Materassi, M. , Neacsu, V. , Beresford‐Webb, J. , Hussin, A. , Khan, N. , Newton, F. , & Galli, G. (2019). Effects of anodal transcranial direct current stimulation over the ventrolateral prefrontal cortex on episodic memory formation and retrieval. Cerebral Cortex, 29, 657–665. 10.1093/cercor/bhx347 29329367

[ejn15649-bib-0081] Mendelsohn, A. , Furman, O. , & Dudai, Y. (2010). Signatures of memory: Brain coactivations during retrieval distinguish correct from incorrect recollection. Frontiers in Behavioral Neuroscience, 4, 18. 10.3389/fnbeh.2010.00018 20428498 PMC2859810

[ejn15649-bib-0082] Meng, A. , Kaiser, M. , Graaf, T. A. , Ducker, F. , Sack, A. T. , De Weerd, P. , & Ven, V. (2021). Transcranial alternating current stimulation at theta frequency to left parietal cortex impairs associative, but not perceptual, memory encoding. Neurobiology of Learning and Memory, 182, 107444. 10.1016/j.nlm.2021.107444 33895350

[ejn15649-bib-0083] Metzler‐Baddeley, C. , Hunt, S. , Jones, D.K. , Leemans, A. , Aggleton, J.P. & OSullivan, M.J.J.N. (2012) Temporal association tracts and the breakdown of episodic memory in mild cognitive impairment. 79, 2233–2240. 23 10.1212/WNL.0b013e31827689e8 PMC3542350 23175726

[ejn15649-bib-0084] Milner, B. (1972). Disorders of learning and memory after temporal lobe lesions in man. Neurosurgery, 19(CN_suppl_1), 421–446. 10.1093/neurosurgery/19.CN_suppl_1.421 4637561

[ejn15649-bib-0085] Mitchell, A. J. , Beaumont, H. , Ferguson, D. , Yadegarfar, M. , & Stubbs, B. (2014). Risk of dementia and mild cognitive impairment in older people with subjective memory complaints: Meta‐analysis. Acta Psychiatrica Scandinavica, 130(6), 439–451. 10.1111/acps.12336 25219393

[ejn15649-bib-0086] Mitchell, K. J. , & Johnson, M. K. (2009). Source monitoring 15 years later: What have we learned from fMRI about the neural mechanisms of source memory? Psychological Bulletin, 135(4), 638–677. 10.1037/a0015849 19586165 PMC2859897

[ejn15649-bib-0087] Montaldi, D. , Spencer, T. J. , Roberts, N. , & Mayes, A. R. (2006). The neural system that mediates familiarity memory. Hippocampus, 16, 504–520. 10.1002/hipo.20178 16634088

[ejn15649-bib-0088] Moritz, S. , Glascher, J. , Sommer, T. , Buchel, C. , & Braus, D. F. (2006). Neural correlates of memory confidence. NeuroImage, 33(4), 1188–1193. 10.1016/j.neuroimage.2006.08.003 17029986

[ejn15649-bib-0089] Mugikura, S. , Abe, N. , Suzuki, M. , Ueno, A. , Higano, S. , Takahashi, S. , & Fujii, T. (2010). Hippocampal activation associated with successful external source monitoring. Neuropsychologia, 48(6), 1543–1550. 10.1016/j.neuropsychologia.2010.01.021 20117120

[ejn15649-bib-0090] Muller, A. , Sirianni, L. A. , & Addante, R. J. (2021). Neural correlates of the dunning–Kruger effect. European Journal of Neuroscience, 53, 460–484. 10.1111/ejn.14935 32761954 PMC7920517

[ejn15649-bib-0091] Muller, J. , & Roberts, J. E. (2005). Memory and attention in obsessive–compulsive disorder: A review. Journal of Anxiety Disorders, 19, 1–28. 10.1016/j.janxdis.2003.12.001 15488365

[ejn15649-bib-0092] Murray, L. J. , & Ranganath, C. (2007). The dorsolateral prefrontal cortex contributes to successful relational memory encoding. The Journal of Neuroscience, 27, 5515–5522. 10.1523/JNEUROSCI.0406-07.2007 17507573 PMC6672342

[ejn15649-bib-0093] Nilakantan, A. S. , Bridge, D. J. , Gagnon, E. P. , VanHaerents, S. A. , & Voss, J. L. (2017). Stimulation of the posterior cortical‐hippocampal network enhances precision of memory recollection. Current Biology, 27(3), 465–470. 10.1016/j.cub.2016.12.042 28111154 PMC5302852

[ejn15649-bib-0094] Nyhus, E. , & Badre, D. (2015). Functional organization of frontal cortex. In The Wiley handbook on the cognitive neuroscience of memory. Wiley.

[ejn15649-bib-0095] Nyhus, E. , & Curran, T. (2010). Functional role of gamma and theta oscillations in episodic memory. Neuroscience & Biobehavioral Reviews, 34(7), 1023–1035. 10.1016/j.neubiorev.2009.12.014 20060015 PMC2856712

[ejn15649-bib-0096] Oijen, M. , Jong, F. J. , Hofman, A. , Koudstaal, P. J. , & Breteler, M. M. (2007). Subjective memory complaints, education, and risk of Alzheimer's disease. Alzheimers Dement, 3, 92–97. 10.1016/j.jalz.2007.01.011 19595922

[ejn15649-bib-0097] Otten, L. J. , Henson, R. N. , & Rugg, M. D. (2001). Depth of processing effects on neural correlates of memory encoding: Relationship between findings from across‐ and within‐task comparisons. Brain, 124, 399–412. 10.1093/brain/124.2.399 11157567

[ejn15649-bib-0098] Otten, L. J. , & Rugg, M. D. (2001). When more means less: Neural activity related to unsuccessful memory encoding. Current Biology, 11(19), 1528–1530. 10.1016/S0960-9822(01)00454-7 11591321

[ejn15649-bib-0099] Pena, M. M. , Klemfuss, J. Z. , Loftus, E. F. , & Mindthoff, A. (2017). The effects of exposure to differing amounts of misinformation and source credibility perception on source monitoring and memory accuracy. Psychology of Consciousness: Theory, Research, and Practice, 4, 337–347. 10.1037/cns0000137

[ejn15649-bib-0100] Preston, A. R. , & Eichenbaum, H. (2013). Interplay of hippocampus and prefrontal cortex in memory. Current Biology, 23, R764–R773. 10.1016/j.cub.2013.05.041 24028960 PMC3789138

[ejn15649-bib-0101] Qin, S. , Marle, H. J. , Hermans, E. J. , & Fernandez, G. (2011). Subjective sense of memory strength and the objective amount of information accurately remembered are related to distinct neural correlates at encoding. The Journal of Neuroscience, 31(24), 8920–8927. 10.1523/JNEUROSCI.2587-10.2011 21677175 PMC6622961

[ejn15649-bib-0102] Qin, S. , Piekema, C. , Petersson, K. M. , Han, B. , Luo, J. , & Fernandez, G. (2007). Probing the transformation of discontinuous associations into episodic memory: An event‐related fMRI study. NeuroImage, 38(1), 212–222. 10.1016/j.neuroimage.2007.07.020 17804259

[ejn15649-bib-0103] Ramanan, S. , Piguet, O. , & Irish, M. (2018). Rethinking the role of the angular gyrus in remembering the past and imagining the future: The contextual integration model. The Neuroscientist, 24(4), 342–352. 10.1177/1073858417735514 29283042

[ejn15649-bib-0104] Ranganath, C. (2010). A unified framework for the functional organization of the medial temporal lobes and the phenomenology of episodic memory. Hippocampus, 20(11), 1263–1290. 10.1002/hipo.20852 20928833

[ejn15649-bib-0105] Reed, C. M. , Mosher, C. P. , Chandravadia, N. , Chung, J. M. , Mamelak, A. N. , & Rutishauser, U. (2020). Extent of single‐neuron activity modulation by hippocampal interictal discharges predicts declarative memory disruption in humans. Journal of Neuroscience, 40(3), 682–693. 10.1523/JNEUROSCI.1380-19.2019 31754015 PMC6961998

[ejn15649-bib-0106] Rey, H. G. , De Falco, E. , Ison, M. J. , Valentin, A. , Alarcon, G. , Selway, R. , Richardson, M. P. , & Quian Quiroga, R. (2018). Encoding of long‐term associations through neural unitization in the human medial temporal lobe. Nature Communications, 9(1), 4372. 10.1038/s41467-018-06870-2 PMC6197188 30348996

[ejn15649-bib-0107] Risius, U. M. , Staniloiu, A. , Piefke, M. , Maderwald, S. , Schulte, F. P. , Brand, M. , & Markowitsch, H. J. (2013). Retrieval, monitoring, and control processes: A 7 tesla FMRI approach to memory accuracy. Frontiers in Behavioral Neuroscience, 7, 24. 10.3389/fnbeh.2013.00024 23580061 PMC3619143

[ejn15649-bib-0108] Ritchey, M. , Libby, L. A. , & Ranganath, C. (2015). Cortico‐hippocampal systems involved in memory and cognition: The PMAT framework. Progress in Brain Research, 219, 45–64. 10.1016/bs.pbr.2015.04.001 26072233

[ejn15649-bib-0109] Rossi, S. , Pasqualetti, P. , Zito, G. , Vecchio, F. , Cappa, S. F. , Miniussi, C. , Babiloni, C. , & Rossini, P. M. (2006). Prefrontal and parietal cortex in human episodic memory: An interference study by repetitive transcranial magnetic stimulation. The European Journal of Neuroscience, 23(3), 793–800. 10.1111/j.1460-9568.2006.04600.x 16487159

[ejn15649-bib-0110] Rubinstein, D. Y. , Camarillo‐Rodriguez, L. , Serruya, M. D. , Herweg, N. A. , Waldman, Z. J. , Wanda, P. A. , Sharan, A. D. , Weiss, S. A. , & Sperling, M. R. (2021). Contribution of left supramarginal and angular gyri to episodic memory encoding: An intracranial EEG study. NeuroImage, 225, 117514. 10.1016/j.neuroimage.2020.117514 33137477

[ejn15649-bib-0111] Rugg, M.D. (2004) Retrieval processing in human memory: Electrophysiological and fMRI evidence.

[ejn15649-bib-0112] Rugg, M. D. , Henson, R. N. , & Robb, W. G. (2003). Neural correlates of retrieval processing in the prefrontal cortex during recognition and exclusion tasks. Neuropsychologia, 41(1), 40–52. 10.1016/S0028-3932(02)00129-X 12427564

[ejn15649-bib-0113] Rugg, M. D. , & Vilberg, K. L. (2013). Brain networks underlying episodic memory retrieval. Current Opinion in Neurobiology, 23, 255–260. 10.1016/j.conb.2012.11.005 23206590 PMC3594562

[ejn15649-bib-0114] Rugg, M. D. , Vilberg, K. L. , Mattson, J. T. , Yu, S. S. , Johnson, J. D. , & Suzuki, M. (2012). Item memory, context memory and the hippocampus: fMRI evidence. Neuropsychologia, 50(13), 3070–3079. 10.1016/j.neuropsychologia.2012.06.004 22732490 PMC3472091

[ejn15649-bib-0115] Rungratsameetaweemana, N. , Squire, L. R. , & Serences, J. T. (2019). Preserved capacity for learning statistical regularities and directing selective attention after hippocampal lesions. Proceedings of the National Academy of Sciences, 116(39), 19705–19710. 10.1073/pnas.1904502116 PMC6765262 31492814

[ejn15649-bib-0116] Rutishauser, U. , Aflalo, T. , Rosario, E. R. , Pouratian, N. , & Andersen, R. A. (2018). Single‐neuron representation of memory strength and recognition confidence in left human posterior parietal cortex. Neuron, 97(1), 209–220. 10.1016/j.neuron.2017.11.029 29249283 PMC5754243

[ejn15649-bib-0117] Sabb, F. W. , Bilder, R. M. , Chou, M. , & Bookheimer, S. Y. (2007). Working memory effects on semantic processing: Priming differences in pars orbitalis. NeuroImage, 37(1), 311–322. 10.1016/j.neuroimage.2007.04.050 17555989

[ejn15649-bib-0118] Schacter, D. L. , & Wagner, A. D. (1999). Medial temporal lobe activations in fMRI and PET studies of episodic encoding and retrieval. Hippocampus, 9, 7–24. 10.1002/(SICI)1098-1063(1999)9:1<7::AID-HIPO2>3.0.CO;2-K 10088896

[ejn15649-bib-0119] Schapiro, A. C. , Gregory, E. , Landau, B. , McCloskey, M. , & Turk‐Browne, N. B. (2014). The necessity of the medial temporal lobe for statistical learning. Journal of Cognitive Neuroscience, 26(8), 1736–1747. 10.1162/jocn_a_00578 24456393 PMC4264662

[ejn15649-bib-0120] Schnyer, D. M. , Verfaellie, M. , Alexander, M. P. , LaFleche, G. , Nicholls, L. , & Kaszniak, A. W. (2004). A role for right medial prefrontal cortex in accurate feeling‐of‐knowing judgments: Evidence from patients with lesions to frontal cortex. Neuropsychologia, 42(7), 957–966. 10.1016/j.neuropsychologia.2003.11.020 14998710

[ejn15649-bib-0121] Scoville, W. B. , & Milner, B. (1957). Loss of recent memory after bilateral hippocampal lesions. Journal of Neurology, Neurosurgery, and Psychiatry, 20, 11–21. 10.1136/jnnp.20.1.11 13406589 PMC497229

[ejn15649-bib-0122] Sestieri, C. , Shulman, G. L. , & Corbetta, M. (2017). The contribution of the human posterior parietal cortex to episodic memory. Nature Reviews. Neuroscience, 18(3), 183–192. 10.1038/nrn.2017.6 28209980 PMC5682023

[ejn15649-bib-0123] Shing, Y. L. , Werkle‐Bergner, M. , Li, S.‐C. , & Lindenberger, U. (2009). Committing memory errors with high confidence: Older adults do but children don't. Memory, 17, 169–179. 10.1080/09658210802190596 18608975

[ejn15649-bib-0124] Shrager, Y. , Kirwan, C. B. , & Squire, L. R. (2008). Activity in both hippocampus and perirhinal cortex predicts the memory strength of subsequently remembered information. Neuron, 59(4), 547–553. 10.1016/j.neuron.2008.07.022 18760691 PMC2614916

[ejn15649-bib-0125] Simons, J. S. , Peers, P. V. , Mazuz, Y. S. , Berryhill, M. E. , & Olson, I. R. (2010). Dissociation between memory accuracy and memory confidence following bilateral parietal lesions. Cerebral Cortex, 20, 479–485. 10.1093/cercor/bhp116 19542474 PMC2803741

[ejn15649-bib-0126] Skinner, E. I. , & Fernandes, M. A. (2007). Neural correlates of recollection and familiarity: A review of neuroimaging and patient data. Neuropsychologia, 45(10), 2163–2179. 10.1016/j.neuropsychologia.2007.03.007 17445844

[ejn15649-bib-0127] Slotnick, S. D. , & Thakral, P. P. (2013). The hippocampus operates in a threshold manner during spatial source memory. Neuroreport, 24(5), 265–269. 10.1097/WNR.0b013e32835f282d 23407277

[ejn15649-bib-0128] Solomon, E. A. , Stein, J. M. , Das, S. , Gorniak, R. , Sperling, M. R. , Worrell, G. , Inman, C. S. , Tan, R. J. , Jobst, B. C. , Rizzuto, D. S. , & Kahana, M. J. (2019). Dynamic theta networks in the human medial temporal lobe support episodic memory. Current Biology, 29(7), 1100–1111. 10.1016/j.cub.2019.02.020 30905609 PMC6445741

[ejn15649-bib-0129] Sommer, T. , Rose, M. , Weiller, C. , & Buchel, C. (2005). Contributions of occipital, parietal and parahippocampal cortex to encoding of object‐location associations. Neuropsychologia, 43(5), 732–743. 10.1016/j.neuropsychologia.2004.08.002 15721186

[ejn15649-bib-0130] Song, Z. , Jeneson, A. , & Squire, L. R. (2011). Medial temporal lobe function and recognition memory: A novel approach to separating the contribution of recollection and familiarity. The Journal of Neuroscience, 31(44), 16026–16032. 10.1523/JNEUROSCI.3012-11.2011 22049444 PMC3227550

[ejn15649-bib-0131] Song, Z. , Wixted, J. T. , Smith, C. N. , & Squire, L. R. (2011). Different nonlinear functions in hippocampus and perirhinal cortex relating functional MRI activity to memory strength. Proceedings of the National Academy of Sciences of the United States of America, 108(14), 5783–5788. 10.1073/pnas.1103225108 21436048 PMC3078356

[ejn15649-bib-0132] Spalding, K. N. , Schlichting, M. L. , Zeithamova, D. , Preston, A. R. , Tranel, D. , Duff, M. C. , & Warren, D. E. (2018). Ventromedial prefrontal cortex is necessary for normal associative inference and memory integration. Journal of Neuroscience, 38(15), 3767–3775. 10.1523/JNEUROSCI.2501-17.2018 29555854 PMC5895999

[ejn15649-bib-0133] Spaniol, J. , Davidson, P. S. , Kim, A. S. , Han, H. , Moscovitch, M. , & Grady, C. L. (2009). Event‐related fMRI studies of episodic encoding and retrieval: Meta‐analyses using activation likelihood estimation. Neuropsychologia, 47(8–9), 1765–1779. 10.1016/j.neuropsychologia.2009.02.028 19428409

[ejn15649-bib-0134] Squire, L. R. , Wixted, J. T. , & Clark, R. E. (2007). Recognition memory and the medial temporal lobe: A new perspective. Nature Reviews Neuroscience, 8(11), 872–883. 10.1038/nrn2154 17948032 PMC2323975

[ejn15649-bib-0135] Staresina, B. P. , Cooper, E. , & Henson, R. N. (2013). Reversible information flow across the medial temporal lobe: The hippocampus links cortical modules during memory retrieval. Journal of Neuroscience, 33(35), 14184–14192. 10.1523/JNEUROSCI.1987-13.2013 23986252 PMC3756762

[ejn15649-bib-0136] Starns, J. J. (2021). High‐and low‐threshold models of the relationship between response time and confidence. Journal of Experimental Psychology: Learning, Memory, and Cognition, 47, 671. 33090841 10.1037/xlm0000960

[ejn15649-bib-0137] Tambini, A. , Nee, D. E. , & D'Esposito, M. (2018). Hippocampal‐targeted theta‐burst stimulation enhances associative memory formation. Journal of Cognitive Neuroscience, 30(10), 1452–1472. 10.1162/jocn_a_01300 29916791 PMC7467684

[ejn15649-bib-0138] Teyler, T. J. , & Rudy, J. W. (2007). The hippocampal indexing theory and episodic memory: Updating the index. Hippocampus, 17(12), 1158–1169. 10.1002/hipo.20350 17696170

[ejn15649-bib-0139] Tibon, R. , Fuhrmann, D. , Levy, D. A. , Simons, J. S. , & Henson, R. N. (2019). Multimodal integration and vividness in the angular gyrus during episodic encoding and retrieval. The Journal of Neuroscience, 39(22), 4365–4374. 10.1523/JNEUROSCI.2102-18.2018 30902869 PMC6538859

[ejn15649-bib-0140] Tubridy, S. , & Davachi, L. (2011). Medial temporal lobe contributions to episodic sequence encoding. Cerebral Cortex, 21, 272–280. 10.1093/cercor/bhq092 20494967 PMC3020579

[ejn15649-bib-0141] Uncapher, M. R. , Hutchinson, J. B. , & Wagner, A. D. (2011). Dissociable effects of top‐down and bottom‐up attention during episodic encoding. The Journal of Neuroscience, 31(35), 12613–12628. 10.1523/JNEUROSCI.0152-11.2011 21880922 PMC3172893

[ejn15649-bib-0142] Uncapher, M. R. , Otten, L. J. , & Rugg, M. D. (2006). Episodic encoding is more than the sum of its parts: An fMRI investigation of multifeatural contextual encoding. Neuron, 52(3), 547–556. 10.1016/j.neuron.2006.08.011 17088219 PMC1687210

[ejn15649-bib-0143] Uncapher, M. R. , & Wagner, A. D. (2009). Posterior parietal cortex and episodic encoding: Insights from fMRI subsequent memory effects and dual‐attention theory. Neurobiology of Learning and Memory, 91, 139–154. 10.1016/j.nlm.2008.10.011 19028591 PMC2814803

[ejn15649-bib-0144] VanElzakker, M. , Fevurly, R. D. , Breindel, T. , & Spencer, R. L. (2008). Environmental novelty is associated with a selective increase in Fos expression in the output elements of the hippocampal formation and the perirhinal cortex. Learning & Memory, 15(12), 899–908. 10.1101/lm.1196508 19050162 PMC2632843

[ejn15649-bib-0145] Vulic, K. , Bjekic, J. , Paunovic, D. , Jovanovic, M. , Milanovic, S. , & Filipovic, S. R. (2021). Theta‐modulated oscillatory transcranial direct current stimulation over posterior parietal cortex improves associative memory. Scientific Reports, 11, 3013. 10.1038/s41598-021-82577-7 33542344 PMC7862221

[ejn15649-bib-0146] Wagner, A. D. , Shannon, B. J. , Kahn, I. , & Buckner, R. L. (2005). Parietal lobe contributions to episodic memory retrieval. Trends in Cognitive Sciences, 9, 445–453. 10.1016/j.tics.2005.07.001 16054861

[ejn15649-bib-0147] Wais, P. E. (2011). Hippocampal signals for strong memory when associative memory is available and when it is not. Hippocampus, 21(1), 9–21. 10.1002/hipo.20716 20014387

[ejn15649-bib-0148] Wais, P. E. , Squire, L. R. , & Wixted, J. T. (2010). In search of recollection and familiarity signals in the hippocampus. Journal of Cognitive Neuroscience, 22, 109–123. 10.1162/jocn.2009.21190 19199424 PMC2888779

[ejn15649-bib-0149] Wang, J. X. , Rogers, L. M. , Gross, E. Z. , Ryals, A. J. , Dokucu, M. E. , Brandstatt, K. L. , Hermiller, M. S. , & Voss, J. L. (2014). Targeted enhancement of cortical‐hippocampal brain networks and associative memory. Science, 345(6200), 1054–1057. 10.1126/science.1252900 25170153 PMC4307924

[ejn15649-bib-0150] Wang, W. C. , Ranganath, C. , & Yonelinas, A. P. (2014). Activity reductions in perirhinal cortex predict conceptual priming and familiarity‐based recognition. Neuropsychologia, 52, 19–26. 10.1016/j.neuropsychologia.2013.10.006 24157537 PMC3923843

[ejn15649-bib-0151] Weidemann, C. T. , & Kahana, M. J. (2016). Assessing recognition memory using confidence ratings and response times. Royal Society Open Science, 3(4), 150670. 10.1098/rsos.150670 27152209 PMC4852632

[ejn15649-bib-0152] Woroch, B. , Konkel, A. , & Gonsalves, B. D. (2019). Activation of stimulus‐specific processing regions at retrieval tracks the strength of relational memory. AIMS Neurosci, 6(4), 250–265. 10.3934/Neuroscience.2019.4.250 32341981 PMC7179353

[ejn15649-bib-0153] Wynn, S. C. , Daselaar, S. M. , Kessels, R. P. , & Schutter, D. J. (2019). The electrophysiology of subjectively perceived memory confidence in relation to recollection and familiarity. Brain and Cognition, 130, 20–27. 10.1016/j.bandc.2018.07.003 30677724

[ejn15649-bib-0154] Wynn, S. C. , Hendriks, M. P. H. , Daselaar, S. M. , Kessels, R. P. C. , & Schutter, D. (2018). The posterior parietal cortex and subjectively perceived confidence during memory retrieval. Learning & Memory, 25(8), 382–389. 10.1101/lm.048033.118 30012883 PMC6049393

[ejn15649-bib-0155] Wynn, S. C. , Kessels, R. P. , & Schutter, D. J. (2020). Electrocortical indices of subjectively perceived confidence in episodic memory. International Journal of Psychophysiology, 151, 18–24. 10.1016/j.ijpsycho.2020.02.007 32057779

[ejn15649-bib-0156] Yonelinas, A. P. (2002). The nature of recollection and familiarity: A review of 30 years of research. Journal of Memory and Language, 46, 441–517. 10.1006/jmla.2002.2864

[ejn15649-bib-0157] Yonelinas, A. P. , Otten, L. J. , Shaw, K. N. , & Rugg, M. D. (2005). Separating the brain regions involved in recollection and familiarity in recognition memory. Journal of Neuroscience, 25(11), 3002–3008. 10.1523/JNEUROSCI.5295-04.2005 15772360 PMC6725129

[ejn15649-bib-0158] Yonelinas, A. P. , & Parks, C. M. (2007). Receiver operating characteristics (ROCs) in recognition memory: A review. Psychological Bulletin, 133(5), 800, 832. 10.1037/0033-2909.133.5.800 17723031

